# Inhibition of MyD88 Signaling Skews Microglia/Macrophage Polarization and Attenuates Neuronal Apoptosis in the Hippocampus After Status Epilepticus in Mice

**DOI:** 10.1007/s13311-018-0653-0

**Published:** 2018-08-15

**Authors:** Jin-Tao Liu, Sheng-Xi Wu, Hua Zhang, Fang Kuang

**Affiliations:** 1 Department of Neurosurgery, Tangdu Hospital, The Fourth Military Medical University, No. 569 Xinsi Road, Xi’an, 710038 China; 2 Institute of Neurosciences, Department of Neurobiology and Collaborative Innovation Center for Brain Science, The Fourth Military Medical University, No. 169, Changle West Road, Xi’an, 710032 China; 3 Department of Orthopedics, The 413th Hospital of the Chinese People’s Liberation Army, Zhoushan, 316000 China

**Keywords:** Microglia/macrophage polarization, Status epilepticus, Hippocampus, MyD88, Neuroinflammation

## Abstract

Inflammation is implicated in epileptogenesis. Activated microglia and macrophages (MG/MΦ) are found in the brains of patients with epilepsy-related diseases and animal models of epilepsy. It is not yet known how the MG/MΦ activation phenotype affects pathological changes in the brain after a single seizure. In this study, we had 2 main purposes: first, to characterize post-status epilepticus (SE) inflammation by tracking MG/MΦ polarization, and, second, to explore the role of an innate immune receptor adaptor protein, namely, myeloid differentiation primary response gene 88 (MyD88), in the induction of SE in a mouse model. A lithium–pilocarpine model of seizure conditions was generated in C57BL/6 mice. The intensity and distribution of MG/MΦ polarization were tracked by fluorescent immunohistochemistry and Western blotting for the polarization markers inducible nitrogen oxygenized synthase, arginase-1, CD163, and mannose receptor. We observed steadily increasing M1 MG/MΦ along with MyD88 signal upregulation after SE in the hippocampi of mice, whereas the M2 marker arginase-1 was localized mainly in astrocytes rather than in MG/MΦ. Inhibition or gene knockout of MyD88 reduced M1 MG/MΦ and gliosis although increasing M2 MG/MΦ in the hippocampi of SE mice. MyD88 inhibition also augmented glutamate transporter 1 expression and reduced N-methyl-D-aspartate receptor NR1 subunit expression in the hippocampus to protect pyramidal neurons from apoptosis. These data suggest that MG/MΦ polarization after SE impacts the pathological outcome of the hippocampus via MyD88 signaling and point to MyD88 as a potential neuroprotective target for epilepsy therapy.

**Electronic supplementary material:**

The online version of this article (10.1007/s13311-018-0653-0) contains supplementary material, which is available to authorized users.

## Introduction

Epilepsy is a central nervous system (CNS) disorder characterized by spontaneous seizures that are caused by abnormal electrical activity of large numbers of neurons in the brain. In patients affected by craniocerebral trauma or intracranial infection, a single seizure can be a harbinger of a gradual increase in seizure probability [1–4]. This apparent facilitation of epileptogenesis may be due to changes in the neuronal microenvironment, such as seizure-induced inflammatory responses and changes in glutamate metabolism [4, 5]. Recent evidence indicates that, following status epilepticus (SE) events, cerebral glia are involved in CNS inflammatory processes in a manner that produces intense surges in neural Ca^2+^ conductance via N-methyl-D-aspartate receptor (NMDA) receptors, which then lead to an abnormal increase in intracellular calcium [6–9]. There is a need to clarify putative post-SE pathological glial changes to improve our understanding of microenvironment regulation within the brain and, ultimately, to inform the development of treatment strategies for epilepsy.

Among CNS glial cells, microglia (MG) are highly dynamic brain-resident mononuclear macrophages (MΦ) that regulate immunological processes such as robust chemotaxis, phagocytosis, and cytokine production. They are known to affect the metabolic activity of neurons and astrocytes under stimulation by pathogens or proinflammatory cytokines [10]. In pathological situations, including epilepsy, circulating MΦ cross the blood–brain barrier to reinforce activated MG in combating pathogens or injury [11]. Both MΦ and MG of systemic origin can be activated in a polarizing manner into extreme pro- or anti-inflammatory states, defined as the M1 and M2 phenotypes, respectively [10, 12]. The M1 phenotype, which correlates with the Th1 designation and thus represents classical activation, is characterized by the expression of inducible nitric oxide synthase (iNOS), proinflammatory cytokine release, and antigen presentation. The M2 phenotype, which correlates with the Th2 designation and thus represents alternative activation, is characterized by increased production of anti-inflammatory cytokines as well as increased expression of several proteins, including arginase-1 (ARG-1); chitinase-3-like protein (YKL-40), found in inflammatory zone-1 (FIZZ1); CD163; and the mannose receptor (MR). MG polarization has been observed in diverse CNS pathologies, including Alzheimer disease [13, 14], stroke [15], traumatic brain injury [16], and spinal cord injury [17]. The classical M1 phenotype has been associated with neurodegeneration and has a tumor-suppressive role [18], whereas the alternative M2 MG/MΦ phenotype has been implicated in neuroprotection and promotion of tissue regeneration [19, 20]. Although some authors question whether MG/MΦ polarization exists [21], the pro- and anti-inflammatory states of MG/MΦ indeed determine the outcomes of many CNS diseases. Therefore, these states may also affect the outcome of epilepsy by regulating MG/MΦ polarization, particularly after seizure.

Seizure is a specific symptom of epilepsy that is caused by synchronized neuron excitation. Neuronal excitotoxicity over a large area produces endogenous damage-associated molecular pattern proteins, such as high mobility group box 1 protein (HMGB-1), which activate MG via TLR4 and other innate immune receptors [7, 22]. The relationship between MG/MΦ polarization and seizure induction has attracted some attention but remains unclear as of yet [23–25]. If MG/MΦ polarization could be documented, regulating MG/MΦ polarization toward anti-inflammation would be a therapeutic strategy to address epilepsy by protecting the CNS environment. Among the ways of regulating MG/MΦ polarization, the protein myeloid differentiation primary response gene 88 (MyD88), which has been implicated in diverse functions [26], is an interesting candidate for mediating MG/MΦ involvement in seizure induction. MyD88 is an adaptor protein that is crucial for the proper responsivity of interleukin (IL)-1 and IL-18, as well as for the signaling mechanisms of nearly all Toll-like receptors (TLRs) except TLR3 in MG and MΦ [22] to mediate the inflammatory response. Notably, we observed previously in rats that blockade of MyD88 early after spinal cord injury protected spinal cord cells from secondary injury [27]. Recent evidence has indicated that MyD88 may influence the direction of MG polarization at the mRNA and protein levels [28, 29]. Recent studies have documented MyD88 upregulation in epilepsy models, and inhibition of MyD88 significantly suppressed seizure and neuronal apoptosis in animal models [30, 31]. However, little is known about whether MG/MΦ polarization through MyD88 signaling is involved in epileptic seizures. Based on previous studies and our preliminary data, we hypothesized that MyD88 signaling could be used as a target for neuroprotection after seizure by skewing MG/MΦ toward an anti-inflammatory state.

To test our hypothesis, we designed this study to examine whether MG/MΦ polarization is involved in SE-induced neuroinflammation and pathological changes in the microenvironment in a lithium–pilocarpine mouse model of epilepsy. We explored the role of MyD88 in post-SE sequelae by examining the hippocampi of MyD88 knockout (KO) mice and mice given intrahippocampal injections of MyD88 inhibitor. Inflammatory processes were followed by fluorescent immunohistochemistry (IHC) and Western blotting experiments across a range of post-SE time points.

## Methods and Materials

### Animals and Procedure

Female C57BL/6 mice (age, 8-10 weeks) were purchased from the Experimental Animal Center of the Fourth Military Medical University, China. MyD88 KO (MyD88^−/−^) mice (*n* = 6) and the corresponding wild-type (WT) C57BL/6 mice (*n* = 6) were purchased from the Model Animal Research Center of Nanjing University. All mice were housed in propylene cages (33 cm × 18 cm × 14 cm) at an ambient temperature of 22 ± 2 °C and relative humidity of 50 ± 10%. The animals were maintained on a 12-h:12-h light/dark cycle and given ad libitum access to pelleted semipurified mouse chow (Solid, Vital Keao Feed Co., Beijing, China) and filtered tap water. All animal experiments were carried out in accordance with the National Institutes of Health Guide for the Care and Use of Laboratory Animals (NIH Publications No. 80-23, revised 1996). This study was approved by the Institutional Animal Care and Use Committee and the Committee of Animal Use for Research and Education of the Fourth Military Medical University. All efforts were made to minimize animal suffering and reduce the number of animals used.

### Lithium–Pilocarpine SE Model

**Fig. 1:**
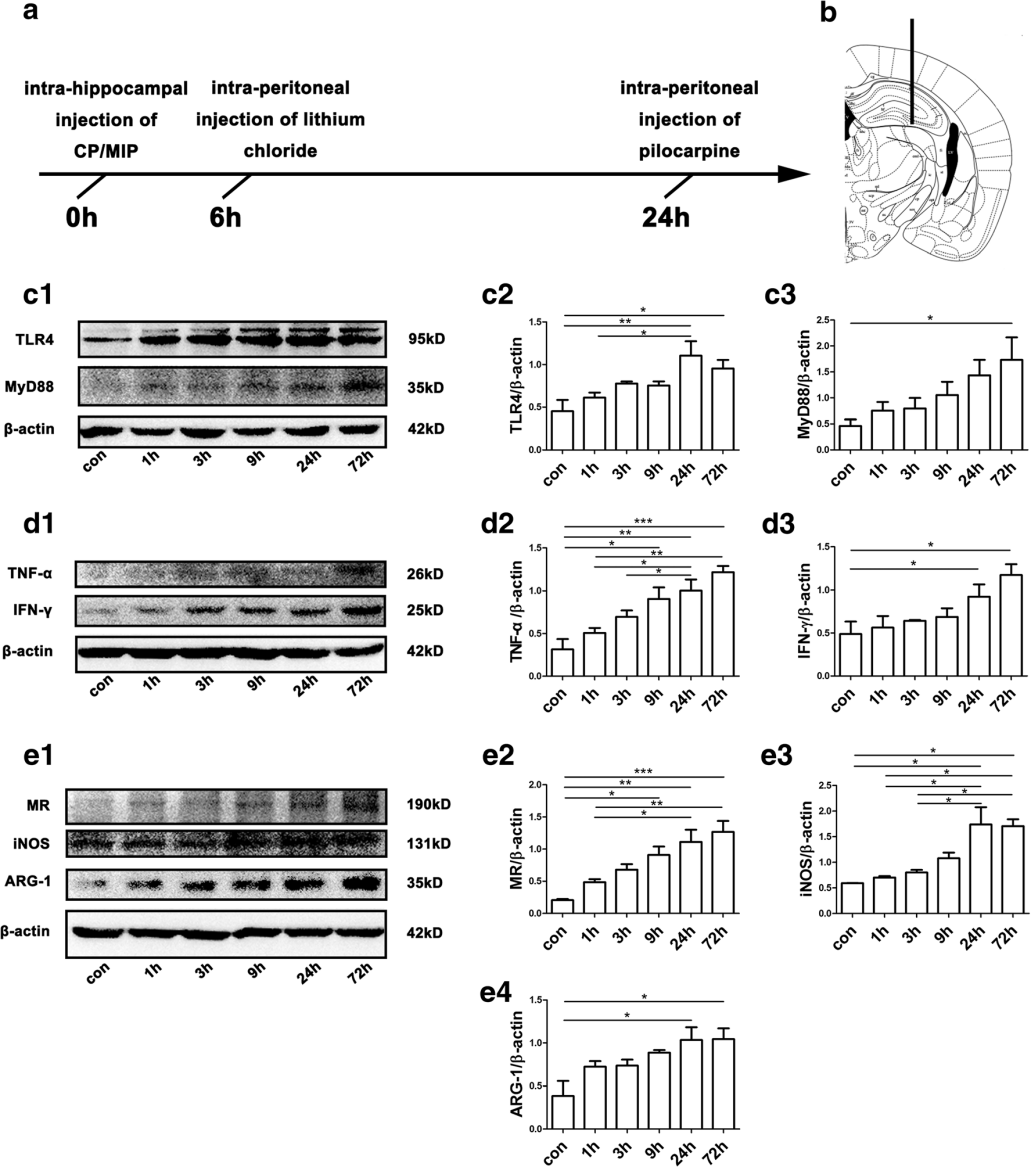
Experimental design and protein levels of markers for M1/M2 polarization and TLR4–MyD88 signaling in mouse hippocampi across time points in the acute phase after SE. (A) Schematic illustration of the experimental procedure. CP or MIP was injected intrahippocampally 6 h prior to an intraperitoneal injection of lithium chloride. Twenty-four hours after the intrahippocampal injection, pilocarpine was administered intraperitoneally. SE was halted by diazepam administration. The mice were sacrificed at different time points after SE, and their brains were collected for analysis. (B) Site of stereotactic unilateral hippocampal injection. (C1) Representative immunoblots illustrate that the abundance of TLR4 and MyD88 in mouse hippocampi increased over time in the acute phase after SE. (C2–C3) Group comparison of TLR4 and MyD88 immunoblots (calibrated relative to β-actin). (D1) Representative immunoblots showing increases in TNF-α and IFN-γ in mouse hippocampi over time up to 72 h after SE. (D2–D3) Group comparison of TNF-α and IFN-γ levels. (E1) Representative immunoblots of MR, iNOS, and ARG-1 for each group; note the relatively rapid increase in iNOS levels over time. (E2–E4) Comparison of MR, iNOS, and ARG-1 levels among the above groups. *N* = 6 per group in the Western blots; *p < 0.05, **p < 0.01, ***p < 0.001 between groups; ANOVA followed by Tukey’s test

A schematic illustration of the epilepsy model is provided in Fig. 1A. Lithium chloride (480 mg/kg, 40 mg/ml, Sigma, 746460, Saint Louis, MO, USA) was injected intraperitoneally 18 to 19 h prior to pilocarpine (96 mg/kg, i.p., 10 mg/ml, Sigma, 1538902, Saint Louis, MO, USA). The subsequent progression of convulsions was evaluated according to a modified version of the Racine scale [32] as follows: grades 1 and 2, facial automatisms, tail stiffening, and wet-dog shakes; grade 3, low-intensity tonic–clonic seizures marked by unilateral forelimb myoclonus in addition to the grade 1/2 symptoms; grade 4, bilateral forelimb myoclonus and rearing; and grade 5, bilateral forelimb and hindlimb myoclonus and transient loss of postural control. After injection of pilocarpine, the mice’s behavior, which consisted generally of salivation, urination, and opisthotonus, was recorded. For this study, the criterion for SE was grade 5 on the aforementioned scale. The first episode of generalized motor convulsions for each mouse was recorded, and those mice that exhibited convulsion of grade 5 were considered successful SE animals. If the first injection of pilocarpine did not induce SE within 30 min, repeated supplemental half-doses of pilocarpine were administered until the mouse reached grade 5. The number of pilocarpine injections was limited to 3 (total dose ≤ 240 mg/kg). SE was not allowed to last longer than 50 min; at the 50-min time point, any ongoing SE was terminated by administering diazepam (10 mg/mg, i.p.). In support of the well being of the animals, each mouse was given a daily 2-ml subcutaneous injection of 0.9% saline containing 1% glucose for 3 days following SE. The mice in the control group were injected with the same doses of saline and diazepam at the same time points as the corresponding SE groups. Comparisons were performed only within each group.

### MyD88 Inhibitory Peptide and Control Peptide Pretreatment

Prior to lithium chloride administration, animals were anesthetized with 7% chloral hydrate and mounted on a stereotaxic frame (RWD Life Science, 68867S, Shenzhen, Guangdong, China) for intracerebral injection. A unilateral intrahippocampal stereotactic injection was administered as shown in Fig. 1B. One microliter of 1 mM control peptide (CP) or MyD88 inhibitory peptide (MIP) (NBP2-29328-5 mg, Novus, Littleton, CO, USA) was injected unilaterally (left side) into the pyramidal cell layer of the hippocampal CA1 (2.0 mm posterior and 1.8 mm lateral to bregma and 2.3 mm ventral to the skull surface [33]) with a Hamilton syringe (5 μl, lot No. 87930, Bonaduz, Switzerland). For the sake of preventing backflow, each 1-μl injection was conducted over a 5-min period, and the injection needle was left in place for an additional 5 min, after which it was withdrawn slowly over another 5-min period. After the injections, the holes in the skulls of the mice were sealed with bone wax. The mice were kept at 37 °C until they awoke from anesthesia (3~4 h).

### Fluorescent IHC

The successfully generated SE models (grade 5, *n* = 3/group) and control animals were sacrificed by transcardial perfusion with 4% paraformaldehyde in 0.1 M phosphate buffer (PB) for 30 min. Their brains were removed and postfixed in 4% paraformaldehyde in PB for 4 h. Subsequently, the brains were equilibrated in 20% sucrose in PB overnight and then transferred to 30% sucrose buffer. Coronal sections (16 μm thick) of the forebrain region containing the hippocampus were collected with a freezing microtome and prepared for fluorescent IHC.

Hippocampal sections were immunolabeled to reveal cellular location and changes in HMGB-1, TLR4, MyD88, ARG-1, CD163, iNOS, MR, iba-1, F4/80, glial fibrillary acidic protein (GFAP), and NeuN. After being blocked in 3% bovine serum albumin (BSA) for 1 h, hippocampal sections were incubated with primary antibody solution containing 0.25% Triton X-100 and 1% BSA at 4 °C overnight. The following primary antibodies were used: rabbit anti-GFAP (Millipore, AB5804, Darmstadt, Germany, 1:1000), mouse anti-GFAP (Millipore, MAB360, Darmstadt, Germany, 1:1000), rat anti-F4/80 (Affymetrix eBioscience, 14-4801, Carlsbad, CA, USA, 1:300), rabbit anti-iba-1 (Wako, ctf4377, Osaka, Japan, 1:1000), rabbit anti-NeuN (Millipore, ABN78, Darmstadt, Germany, 1:1000), mouse anti-NeuN (Millipore, MAB377, Darmstadt, Germany, 1:1000), goat anti-ARG-1 (Santa Cruz, sc-18354, Dallas, Texas, USA, 1:200), mouse anti-iNOS (Abcam, ab49999, Cambridge, MA, USA, 1:500), mouse anti-TLR4 (Abcam, ab30667, Cambridge, MA, USA, 1:500), mouse anti-MyD88 (R&D Systems, MAB3109, Minneapolis, MN, USA, 5 μg/ml), mouse anti-HMGB-1 (Novus, NBP2-27396, Littleton, CO, USA, 5 μg/ml), rabbit anti-MR (Abcam, ab64693, Cambridge, MA, USA, 1:500), rabbit anti-GLT-1 (Novus, NBP1-20136, Littleton, CO, USA, 1:100), and rabbit anti-CD163 (Abcam, ab182422, Cambridge, MA, USA, 1:100). After the primary antibody incubation, sections were washed with 0.01 M phosphate-buffered saline (PBS) 3 times for 5 min each and then incubated with secondary antibody at room temperature for 4 h. After 3 additional PBS washes, the sections were counterstained with Hoechst (Sigma, Saint Louis, MO, USA, 1:5000) for 5 min and covered with fluorescence-preserving VECTASHIELD mounting medium (Vector, H-1000, Shanghai, China). Images were captured with a laser scanning confocal microscope (FV-1000; Olympus, Japan).

### Terminal Deoxynucleotidyl Transferase dUTP Nick End Labeling

For detection of apoptotic cells, 3 fixed mouse brains collected at 3 days after SE were sectioned with a cryostat. The sections were processed according to the instructions of the Cell Death Detection Kit (Roche Molecular Biochemicals, Indianapolis, IN, USA). Images were captured with the same laser scanning confocal microscope introduced above.

### Cell Counting for Fluorescent Labeling

Images of interest were analyzed with ImageJ and Image Tool software. The density of positively labeled cells in the dentate gyrus (DG), CA1, and CA3 regions of the hippocampus was calculated based on cell counts. For each animal, 3 levels of the hippocampal sections were chosen for each brain, approximately interaural 2.34 mm, bregma − 1.46 mm; interaural 1.86 mm, bregma − 1.94 mm; and interaural 1.34 mm, bregma − 2.46 mm, according to Paxinos and Franklin’s mouse brain atlas [33]. The dorsal hippocampus of these levels was divided as CA1, CA3 (including CA2), and DG subareas manually, and the double-stained cells in these levels were judged by eye. Then, the area was evaluated, and the double-labeled cells were counted using Image software. The final cell density was calculated as the number of cells divided by the area.

### Western Blot Analysis

Mice (*n* = 6 at each time point or each group for analysis) were perfused rapidly and systemically with saline, and then their fresh hippocampi were isolated. The fresh hippocampi were frozen quickly in liquid nitrogen (− 196 °C). The tissue was treated with RIPA lysis buffer (1 g:10 ml) consisting of 50 mM Tris, pH 7.4, 150 mM NaCl, 5 mM EDTA, 0.1% SDS, 1% NP-40, 1% deoxycholate, 1% Triton X-100, 10 mM PMSF, 0.1% proteinase inhibitor, and 0.1% phosphatase inhibitor. The lysates were incubated in ice for 10 min and cleared by spinning in a centrifuge (15 min, 12,000 rpm, 4 °C). After protein content analysis by the Bradford method, the resultant supernatants were mixed 4:1 with sample buffer and then boiled. Equivalent amounts of protein were loaded and separated in individual lanes of an SDS-PAGE gel and then transferred to a polyvinylidene fluoride membrane. The membranes were blocked with tris-buffered saline with Tween (TBS-T; 5% low-fat milk, 20 mM Tris-Cl, pH 7.6, 137 mM NaCl, 0.1% Tween 20; for phosphorylated proteins, we used 5% BSA rather than low-fat milk) for 1 h at room temperature and then incubated overnight at 4 °C with the following primary antibodies: rabbit anti-ARG-1 (Sigma, AV45672, Saint Louis, MO, USA, 1:200), rabbit anti-MR (Abcam, ab64693, Cambridge, MA, USA, 1:1000), mouse anti-iNOS (Abcam, ab49999, Cambridge, MA, USA, 1:500), rabbit anti-MyD88 (Abcam, ab2064, Cambridge, MA, USA, 1:500), mouse anti-TLR4 (Abgent, AP1504a, San Diego, CA, USA, 1:500), rabbit anti-interferon (IFN)-γ (Abcam, ab133566, Cambridge, MA, USA, 1:1000), rabbit anti-tumor necrosis factor (TNF)-α (Abcam, ab6671, Cambridge, MA, USA, 1:200), and rabbit anti-β-actin (Huabio, R1207-1, Hangzhou, Zhejiang, China, 1:4000). After a wash in TBS-T, the sections were incubated in goat anti-rabbit or goat anti-mouse secondary antibody solutions, as appropriate. After another TBS-T wash, the immunoreactive protein bands were visualized with a chemiluminescence gel imaging system (Aplegen, Pleasanton, CA, USA, Omega Lum G, 81-12100-00, Customizable Omega Lum G Image Capture Software). The immunoblot protein bands were measured by densitometry using Gel-Pro Analyzer software. Western blot bands were analyzed by comparison with an internal control (i.e., β-actin), and the data are expressed as the means ± standard errors of the mean (SEM).

### Statistical Analyses

Data from at least 3 animals per group or time point were used in the analyses. The data are presented as the means ± SEM. When 1-way analysis of variance (ANOVA) showed significant differences among means, pairwise comparisons of the means were performed with Tukey’s test. The significance level was set at a *p* value of less than 0.05.

## Results

### Acute Inflammatory Response to SE Produced Mainly by M1 MG/MΦ with Astrogliosis

**Fig. 2:**
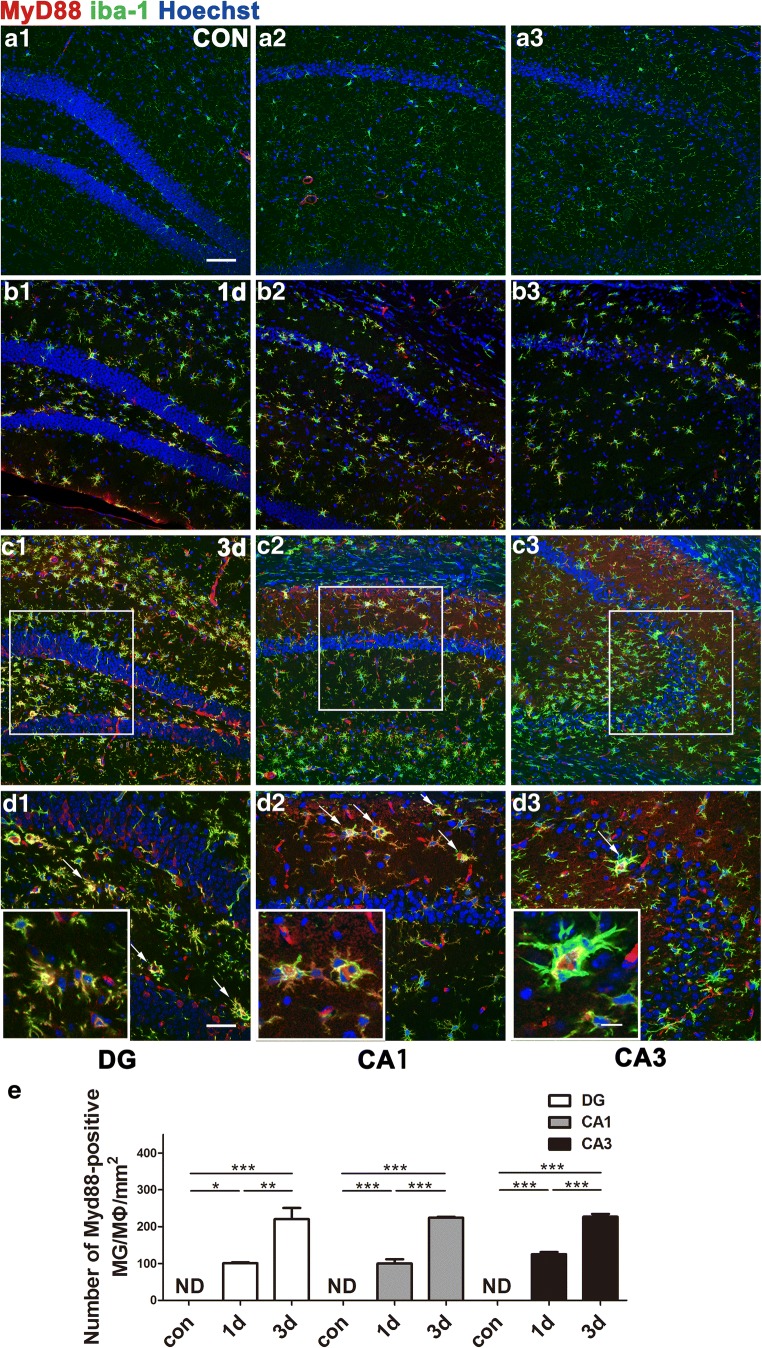
Representative pictures of MyD88-immunoreactive MG/MΦ and their distribution in the DG, CA1, and CA3 of the hippocampus. (A1–A3) Sections from mice in the control group showed neither activated MG/MΦ nor MyD88-positive cells in these regions. Sections from mice at 1 day (B1–B3) and 3 days (C1–C3) after SE showed increased expression of MyD88 specifically in MG/MΦ (identified by iba-1 immunoreactivity). (D1–D3) Higher magnification of the boxes in (C1–C3). Arrows show cells with strong MyD88 and iba-1 immunoreactivity. The insets of (D1, D2, and D3) show additional high-magnification images of MyD88-positive MG/MΦ. (E) Quantitative analysis of iba-1/MyD88 double-labeled cells in hippocampi of control group mice 1 or 3 days after SE (means ± SEM, *n* = 3). *p < 0.05, **p < 0.01, ***p < 0.001 between groups. One-way ANOVA followed by Tukey’s test. ND = not detectable. *Scale bars:* (A1–C3) 100 μm; (D1–D3) 50 μm; (D3) (inset) 12.5 μm

Hippocampal expression level of inflammatory factors in the acute phase following pilocarpine-induced SE was elevated gradually from hour 1 to hour 72. Levels of iNOS protein, a key proinflammatory state marker, were dramatically upregulated 24 h after SE. Meanwhile, the anti-inflammatory state markers ARG-1 and MR protein showed gradual increases. Western blot assays further showed simultaneous activation of TLR4–MyD88 signaling (Fig. 1C–E). MyD88 immunoreactivity was colocalized with that of iba-1, an MG/MΦ marker, throughout the hippocampus, including in the DG, CA1, and CA3. The number of iba-1/MyD88 double-labeled cells was increased (*p* < 0.001) over time after SE compared with that in control mice (Fig. 2). Similarly, TLR4 immunoreactivity was also found mainly in iba-1-positive cells, although some other non-MG/MΦ cells also expressed TLR4 (Supplementary Fig. 1). We observed a dramatic increase in iNOS-immunopositive MG/MΦ in the hippocampi of mice after SE (Supplementary Fig. 2), whereas MG/MΦ immunopositive for ARG-1 or CD163 were few in number (Supplementary Fig. 3 and Supplementary Fig. 4). Conversely, ARG-1 and MR immunolabeling was found predominantly in astrocytes 1~3 days after SE, indicating that ARG-1 and MR upregulation is mediated primarily by astrocytes rather than MG/MΦ (Supplementary Fig. 3 and Supplementary Fig. 5). MyD88 immunolabeling was observed in iba-1-positive cells but seldom found in astrocytes or neurons (Supplementary Fig. 3). We also found increased numbers of ARG-1-immunopositive, activated astrocytes in the hippocampus 3 days after SE (Supplementary Fig. 5).

### M1 MG/MΦ Response and Astrogliosis Were Aggravated in the Acute to Early Chronic Phases After Pilocarpine-Induced SE

**Fig. 3:**
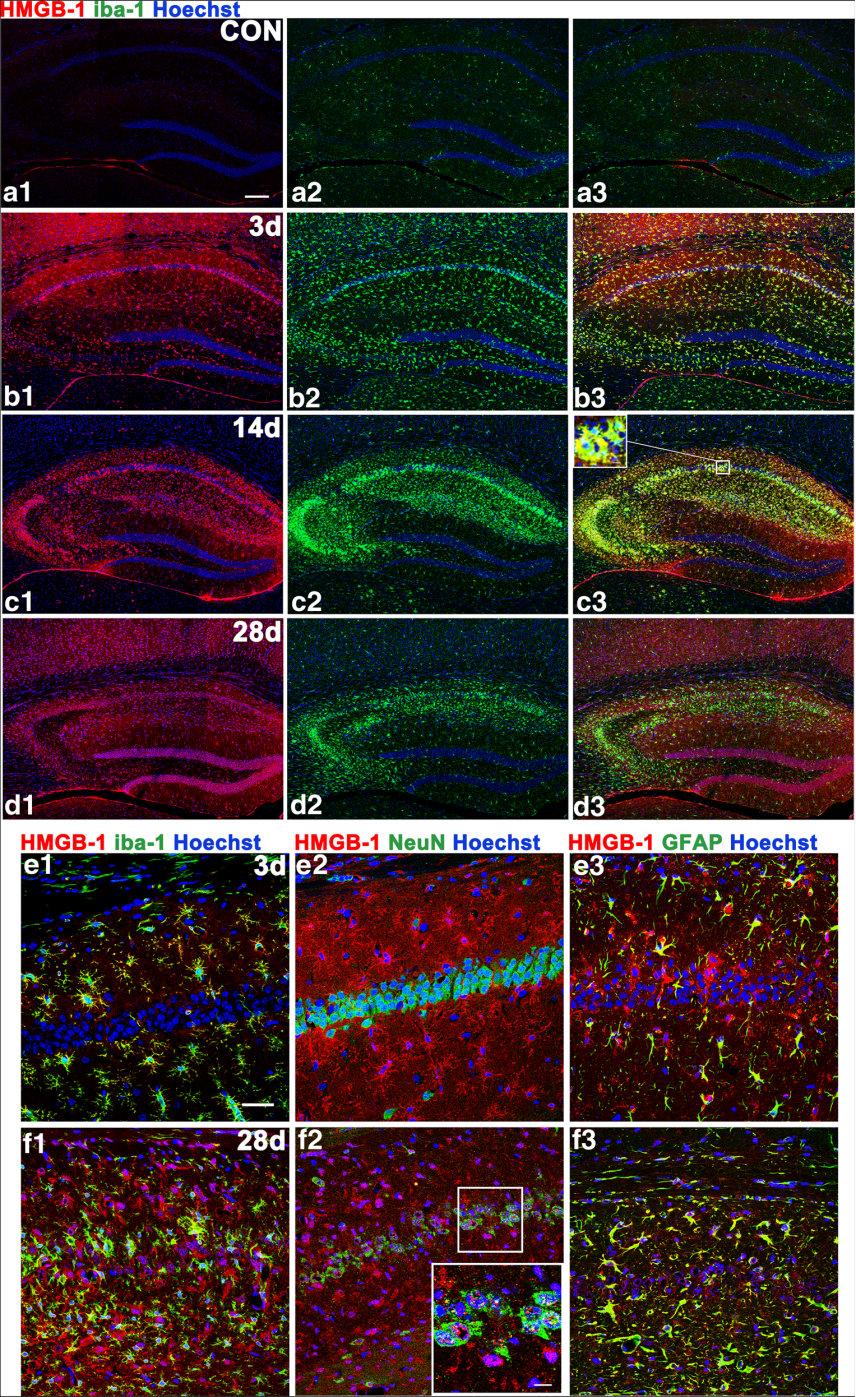
Hippocampal expression of HMGB-1 at various time points and in different cell types. In control animals, there were hardly any HMGB-1-immunoreactive cells. HMGB-1 labeling was intense at 14 days after SE and remained to some extent at 28 days. In the acute phase, the HMGB-1-immunoreactive product was found mainly in the cytoplasm of MG/MΦ and astrocytes. In the chronic phase (28 days), HMGB-1 immunoreactivity was also observed in CA1 pyramidal neurons undergoing karyorrhexis. (A1–D3) The distribution of HMGB-1-positive MG/MΦ in the hippocampi of control animals (A1–A3) at 3 days (B1–B3), 14 days (C1–C3), and 28 days (D1–D3) after SE. The inset of (C3) shows strong HMGB-1 labeling in MG/MΦ. (E1–F3) Colocalization of HMGB-1 immunoreactivity with cell-type markers (NeuN for neurons, GFAP for astrocytes, and iba-1 for MG/MΦ) in CA1 at 3 and 28 days. The inset of (F2) shows a higher-magnification view of HMGB-1-labeled karyorrhectic CA1 pyramidal neurons. (A1–B1–C1–D1), HMGB-1 labeling (red); (A2–B2–C2–D2), iba-1 labeling (green); (A3–B3–C3–D3) merged images. *Scale bars*: (A1–D3) 200 μm; (C) (inset) 12.5 μm; (E1–F3) 50 μm; (F2) (inset) 12.5 μm

HMGB-1 immunoreactivity was elevated in activated MG/MΦ 3 days after SE, remained high 14 days after SE, and continued to be detected at a lower level in the chronic phase (Fig. 3A–D). Note that on day 3 after SE, HMGB-1 was highly expressed in MG/MΦ in particular, with lower expression in astrocytes and hardly any in neurons. By 28 days after SE, that is, in the chronic phase, the nuclei of pyramidal neurons in area CA1 were disintegrated but HMGB-1-positive, and more astrocytes were found to be overexpressing HMGB-1 at this time than in the acute phase (Fig. 3E–F).

**Fig. 4:**
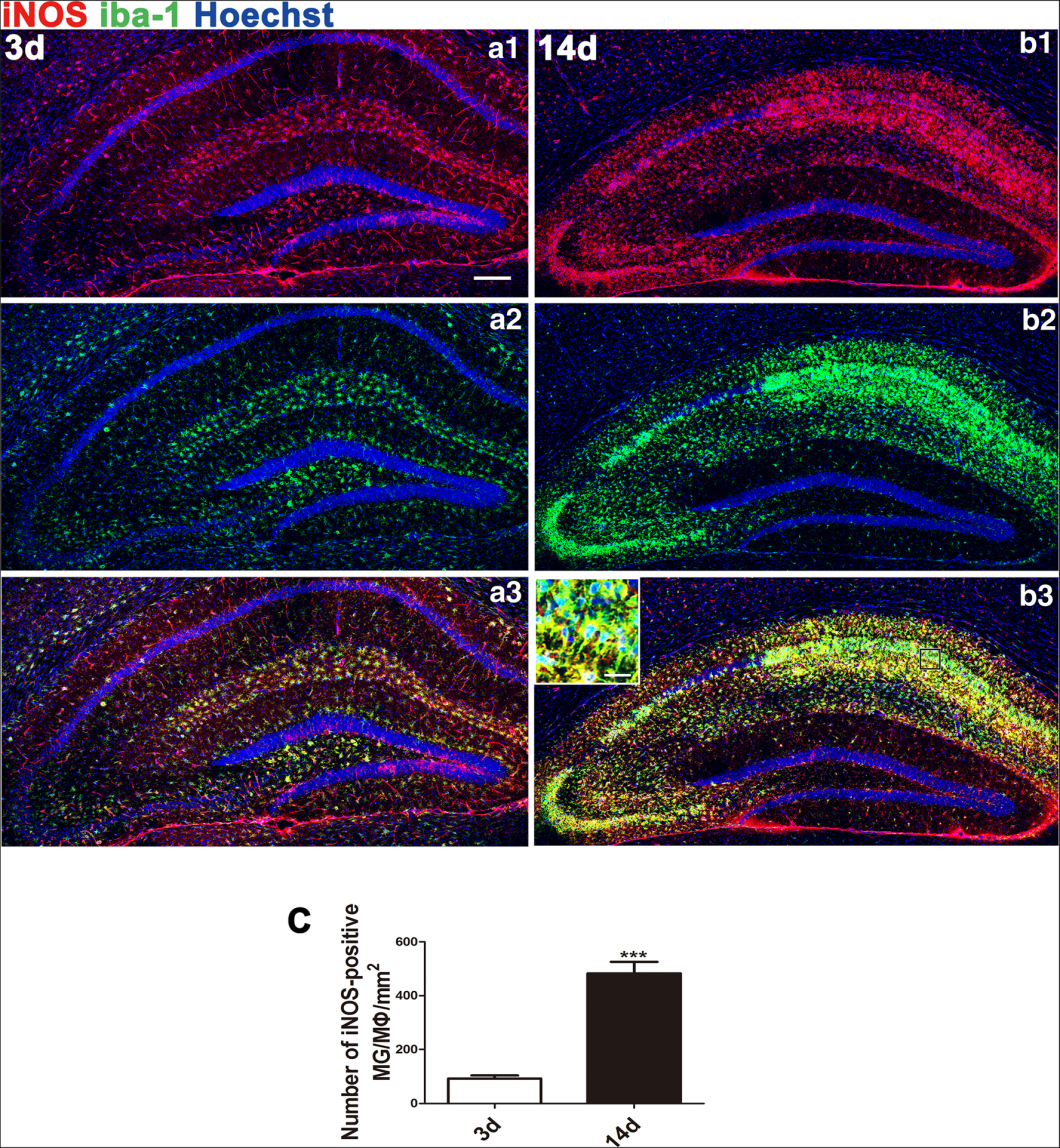
Distribution of iNOS/iba-1 double-labeled cells in mouse hippocampi 3 and 14 days after SE. Cells that stained positive for both iNOS (M1) and the MG/MΦ marker iba-1 were dramatically increased from the acute phase to the early chronic phase throughout the hippocampus with regional changes in distribution. M1 MG/MΦ were distributed in the hippocampi 3 days (A1–A3) and 14 days (B1–B3) after SE. At 3 days, M1 MG/MΦ were gathered mainly in the stratum lacunosum-moleculare of the CA1 and the hilus of the hippocampus, with some scattered outside the hippocampus, including in the cerebral cortex and the dorsal thalamus. At 14 days, high numbers of M1 MG/MΦ were found in areas CA1, CA2, CA3, and CA4 (especially in the stratum pyramidale and stratum radiatum), but M1 MG/MΦ were rarely observed in the DG or outside the hippocampus. The inset in (B3) shows a higher-magnification view of M1 MG/MΦ. (C) Quantitative analysis of iba-1/iNOS double-labeled cells in hippocampi at 3 and 14 days after SE (means ± SEM, *n* = 3). Independent samples *t* test; ***p < 0.001 between groups. *Scale bars*: (A1–C) 200 μm; (B3) (inset) 12.5 μm

We also observed a significant increase in M1 MG/MΦ at 14 days compared with 3 days after SE. At 3 days after SE, M1 MG/MΦ were concentrated in the stratum lacunosum-moleculare of area CA1 and the hilus of the hippocampus and scattered outside of the hippocampus, particularly in the cerebral cortex and hypothalamus. By 14 days after SE, the distribution of M1 MG/MΦ changed, becoming most prevalent in areas CA1 (mainly occupying the stratum oriens, the stratum radiatum, and especially the stratum pyramidale), CA2, and CA3 (stratum pyramidale), while remaining rare in the DG and outside the hippocampus (Fig. 4).

### MyD88 Deficiency or Inhibition Blunted SE Induction and Reduced the Number of Apoptotic Neurons and M1 MG/MΦ but Increased the Number of M2 MG/MΦ in the Hippocampus

**Fig. 5:**
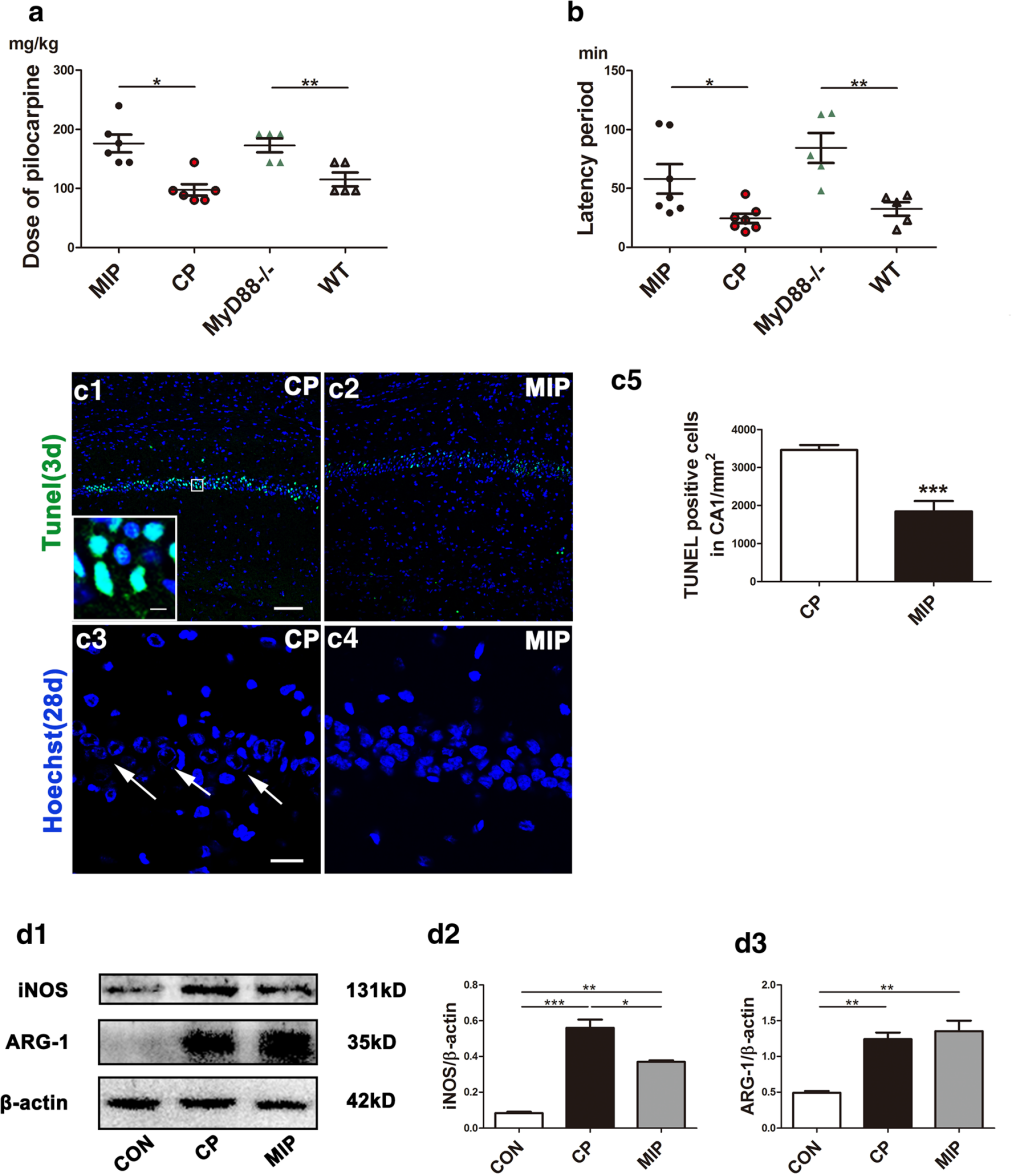
Effects of inhibition of MyD88 on SE occurrence, pyramidal neuron survival in mouse hippocampi, and expression of MG/MΦ polarization markers. (A) Comparison of the pilocarpine dose necessary to induce SE between the MIP and CP groups and between the MyD88^−/−^ and WT groups. (B) Comparison of the latency period of SE between the MIP and CP groups and between the MyD88^−/−^ and WT groups. Means ± SEM; *n* = 5; *p < 0.05 *versus* the CP group/WT group; **p < 0.01 *versus* the CP group/WT group. Independent samples *t* tests were performed. TUNEL staining showing CA1 in the CP (C1) and MIP (C2) groups 3 days after SE. The inset in (C1) shows a high-magnification view of TUNEL-positive CA1 pyramidal neurons. (C3) Remarkably reduced Hoechst staining in area CA1 of the hippocampus in the CP group 28 days after SE. (C4) Hoechst staining showed comparatively normal cell nuclei in the MIP group. Arrows in (C3) indicate cells in karyorrhexis in the CP group. (C5) Quantitative analysis of TUNEL-stained cells in the pyramidal layer of CA1 3 days after SE between the MIP and CP groups. Independent samples *t* test, ***p < 0.001. *Scale bars*: (C1, C2) 100 μm; (C3, C4) 50 μm; (C1) (inset) 12.5 μm. (D1) Immunoblots of iNOS and ARG-1 in the control and the CP and MIP groups’ hippocampi 3 days after SE. (D2–D3) Group comparison of iNOS and ARG-1 levels calibrated to β-actin. *p < 0.05, **p < 0.01, ***p < 0.001 between groups; 1-way ANOVA followed by Tukey’s test

To determine whether M1 polarization is MyD88-dependent, we observed the expression of M1 and M2 markers in the brains of MyD88^−/−^ and MIP-treated mice after SE. During SE induction, we found that MIP-injected and MyD88^−/−^ mice required a significantly higher dose of pilocarpine and a longer period of time (latency) than WT or CP-injected mice to induce SE (Fig. 5A, B). The mortality during seizure induction was 1/6 for MyD88^−/−^ mice and 2/6 for WT mice, whereas the mortality for the MIP and CP groups was 6/40 and 10/40, respectively.

**Fig. 6:**
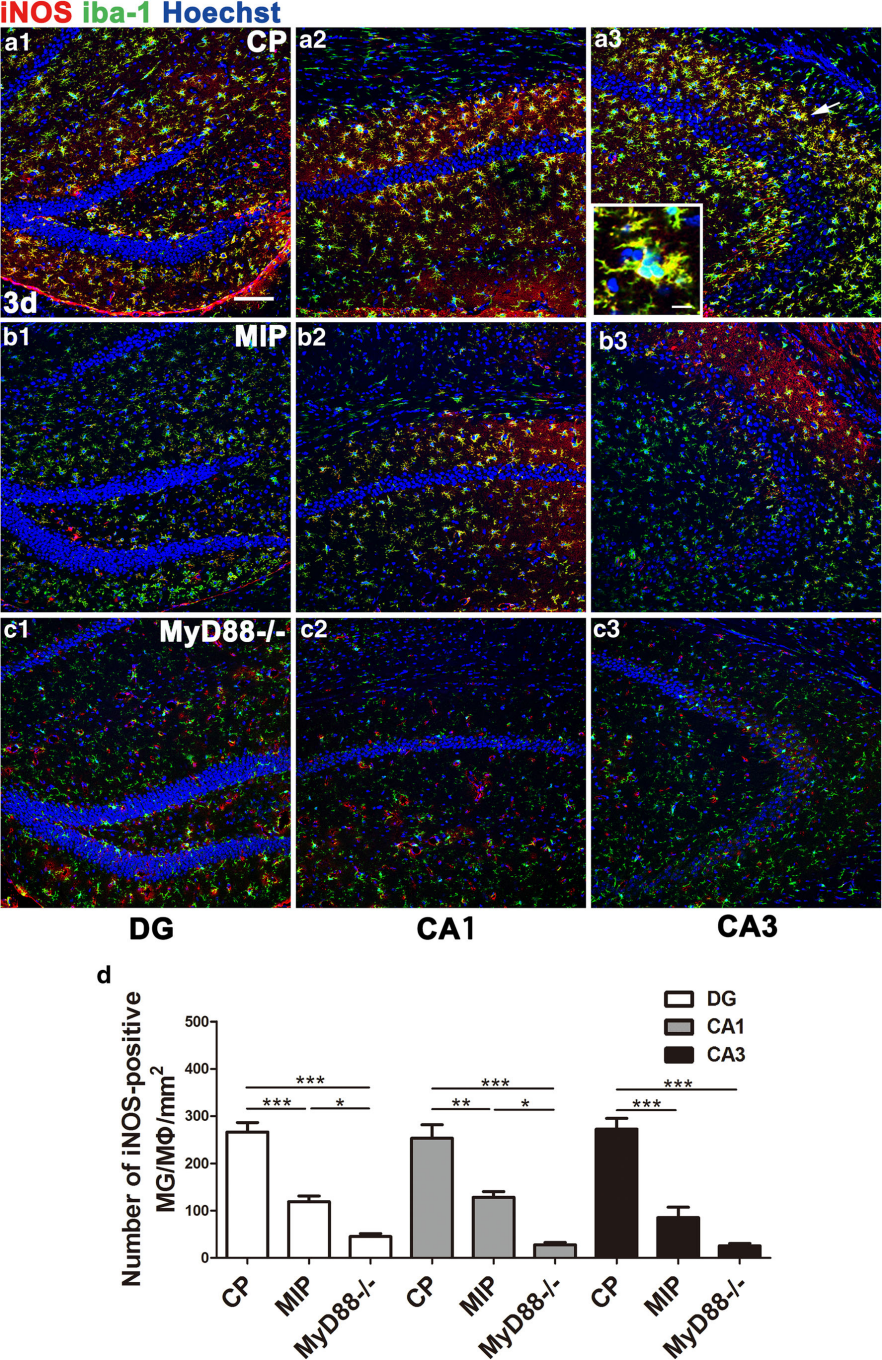
Effect of MyD88 inhibition and MyD88 deficiency on the numbers and distribution of M1 MG/MΦ in the hippocampus 3 days after SE. Immunofluorescence microscopy showed iNOS-positive MG/MΦ in the DG, CA1, and CA3 of mice in the CP group (A1–A3), MIP group (B1–B3), and MyD88^−/−^ group (C1–C3) at 3 days. The inset of (A3) shows a high-magnification view of iNOS and iba-1 double-labeled cells (indicated by arrows) in the CA3. The colocalization of iNOS and iba-1 indicated in yellow shows that iNOS labeling appears in nearly the entire MG/MΦ, including the processes and somata. (D) Group comparison of the numbers of iNOS/iba-1 double-positive cells in the DG, CA1, and CA3 (means ± SEM, *n* = 3). *p < 0.05, **p < 0.01, ***p < 0.001 between groups; 1-way ANOVA followed by Tukey’s test. *Scale bars*: (A1–C3) 100 μm; (A3) (inset) 12.5 μm

**Fig. 7:**
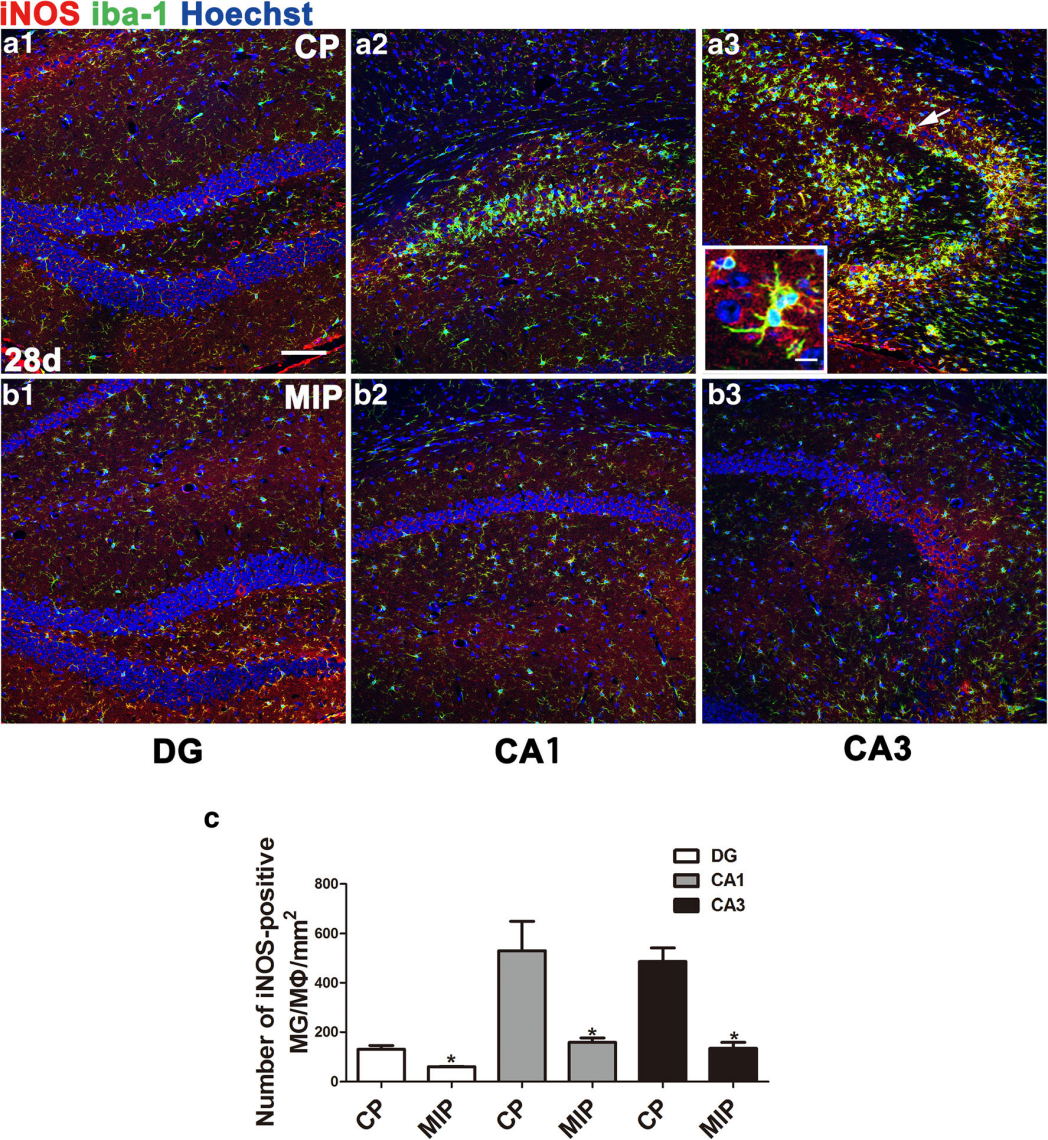
Effect of inhibition of MyD88 on M1 MG/MΦ polarization and gliosis in the chronic phase. iNOS-positive MG/MΦ in the CP group (A1–A3) and MIP group (B1–B3) 28 days after SE. The inset of (A3) shows a high magnification of iNOS and iba-1 double-labeled cells in the CA3 area of the CP group. Note that both iNOS and iba-1 staining could be seen clearly in the processes and soma of the cells. (C) Comparison of the numbers of iba-1/iNOS double-positive cells in the DG, CA1, and CA3 between the CP and MIP groups at the 28-day post-SE time point (means ± SEM, *n* = 3). *p < 0.05 *versus* the CP group; **p < 0.01 *versus* the CP group; ***p < 0.001 *versus* the CP group. Independent samples *t* tests were performed. *Scale bars*: (A1–B3) 100 μm; inset 12.5 μm

Terminal deoxynucleotidyl transferase dUTP nick end labeling (TUNEL) staining (3 days after SE) and nuclear staining (28 days after SE) indicated, respectively, that the MIP treatment reduced apoptosis of CA1 neurons in the acute phase and rescued normal nuclear morphology of neurons in the chronic phase (Fig. 5C). Western blots showed lower hippocampal iNOS levels in MIP-injected mice than in CP-injected mice, but iNOS was not lower in the MIP group than in the control group, which had the lowest level of iNOS among the 3 groups. Similarly, the ARG-1 protein level was extremely low in the normal control group but was significantly elevated in the CP group. The ARG-1 level was slightly higher in the MIP group than in the CP group but not significantly higher in the statistical analysis than in the semiquantitative analysis of immunoblots (Fig. 5D). The number of M1 MG/MΦ was significantly decreased across the hippocampi of MIP-treated animals compared with the CP group. Typical M1 MG/MΦ were not observed in MyD88^−/−^ mice, and few MG/MΦ expressed only low-intensity iNOS immunopositivity (Fig. 6). At 28 days after SE, during the chronic phase, these inhibitory effects of MIP on M1 MG/MΦ were more obvious, and the numbers of M1 MG/MΦ were significantly decreased in all hippocampal subareas of MIP-treated animals compared with those of the CP group (Fig. 7).

**Fig. 8:**
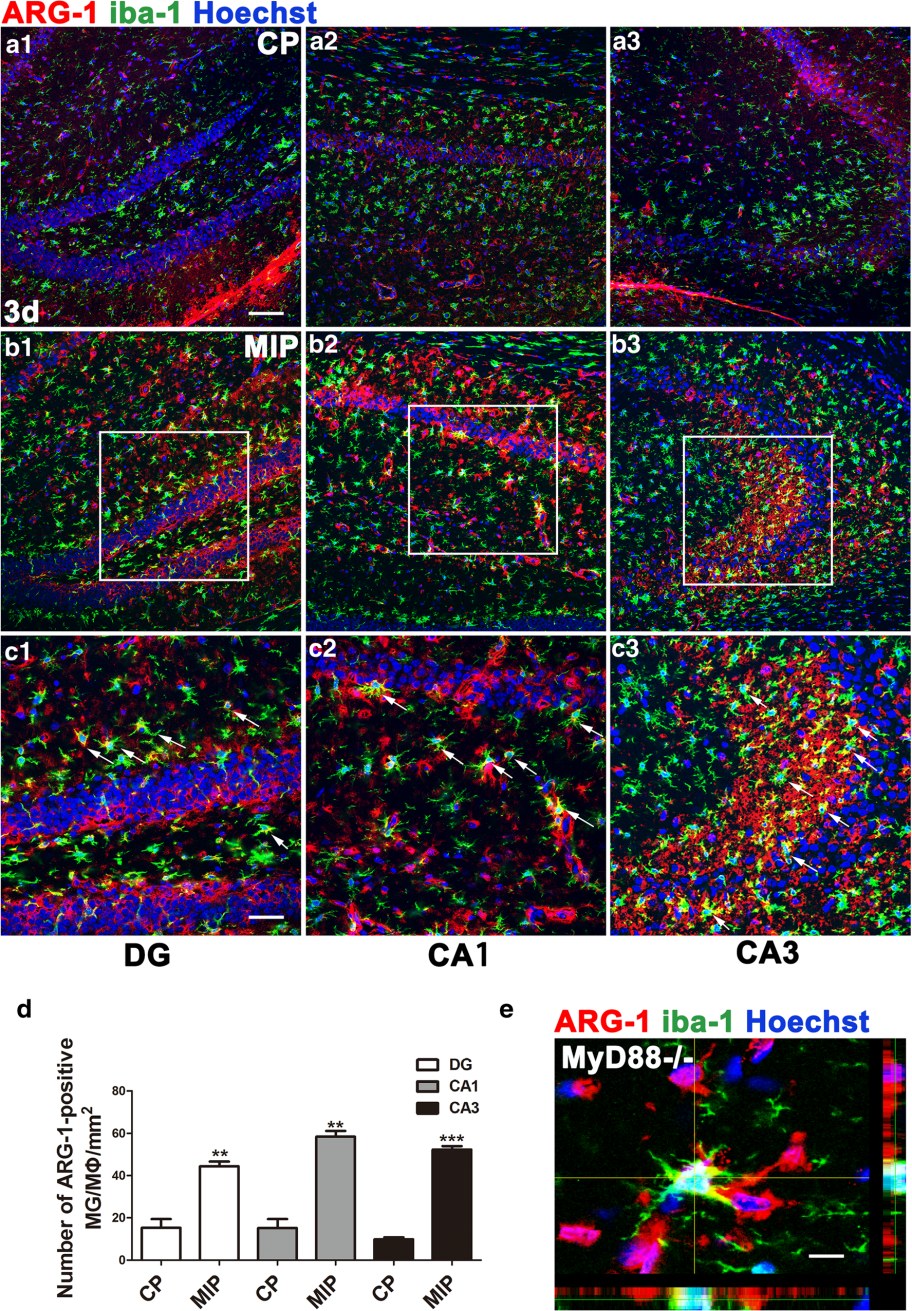
ARG-1 immunolabeled MG/MΦ in the hippocampi of MyD88-inhibited and MyD88-deficient mice 3 days after SE. ARG-1-positive MG/MΦ in the CP group (A1–A3) and MIP group (B1–B3) at 3 days. (C1–C3) Higher magnification of the boxes in (B1–B3). Arrows indicate colocalization of ARG-1 and iba-1 immunoreactivity. (D) Comparison of the numbers of iba-1/ARG-1 double-positive cells in the DG, CA1, and CA3 between the CP and MIP groups (means ± SEM, *n* = 3). **p < 0.01 *versus* the CP group; ***p < 0.001 *versus* the CP group. Independent samples *t* tests were performed. (E) Stack scanning of an ARG-1-positive MG/MΦ in the hippocampus of an MyD88^−/−^ mouse showing colocalization of ARG-1 and iba-1 in the cytoplasm. *Scale bars*: (A1–B3) 100 μm; (C1–C3) 50 μm; (E) 12.5 μm

Both MyD88 inhibition and deficiency (i.e., KO) induced strong ARG-1 expression, typical of M2 morphology, in MG/MΦ in the acute phase (3 days after SE), and the number of ARG-1 and iba-1 double-labeled cells was significantly increased in the hippocampi of MIP and MyD88 KO animals (Fig. 8). ARG-1-positive MG/MΦ were still observable throughout the hippocampus in the chronic phase, 28 days after SE, while microgliosis appeared to be suppressed by MIP treatment (Supplementary Fig. 6). MG/MΦ positive for another M2 marker, CD163, significantly increased in number in the hippocampus of SE mice treated with MIP compared with the mice treated with CP (Supplementary Fig. 4).

### Inhibition of MyD88 Reduced NR1 Expression and Increased GLT-1 Expression

**Fig. 9:**
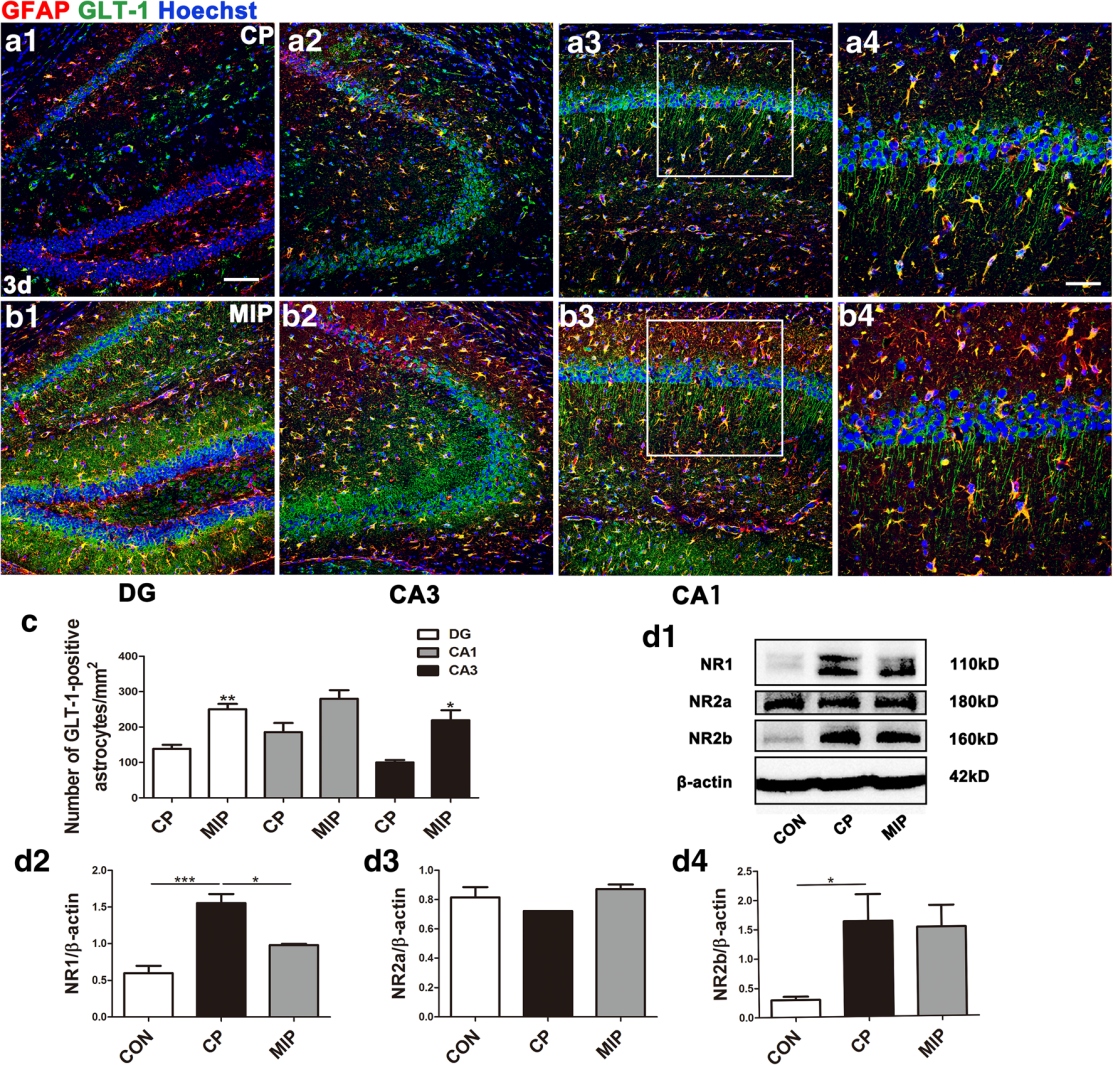
Hippocampal GLT-1 and NR1 expression 3 days after SE with MyD88 inhibition. Sections from the hippocampi of mice in the CP group (A1–A3) and MIP group (B1–B3) 3 days after SE with GLT-1 immunoreactivity in astrocytes and neuronal processes. (A4, B4) Higher magnification of the boxes in (A3) and (B3). (C) Comparison of the numbers of GFAP/GLT-1 double-labeled cells in the DG, CA1, and CA3 between the CP and MIP groups (means ± SEM, *n* = 3). *p < 0.05 *versus* the CP group; **p < 0.01 *versus* the CP group. Independent samples *t* tests were performed. (D1) Immunoblots of NR1, NR2a, and NR2b for the control, CP, and MIP groups. (D2–D4) Comparison of NR1, NR2a, and NR2b levels among the above groups (calibrated to β-actin). *p < 0.05; ***p < 0.001 between groups. One-way ANOVA followed by Tukey’s test. *Scale bars*: (A1–A3, B1–B3) 100 μm; (A4, B4) 50 μm

To investigate the potential protective mechanism of MyD88 inhibition, we used Western blots to detect the expression of glutamate receptor/transporter proteins. We found that hippocampal NR1 and NR2b were increased 3 days after SE, whereas NR2a expression was unchanged. Inhibition of MyD88 reduced NR1 expression but did not affect NR2b or NR2a expression. Fluorescent IHC showed that inhibition of MyD88 in the acute phase increased the number of GLT-1-positive astrocytes, especially in area CA3. MIP treatment also increased neuronal expression of GLT-1, especially in the neurites of pyramidal neurons (Fig. 9).

## Discussion

In this study, using a pilocarpine mouse model, we observed a progressive exacerbation of inflammation in the hippocampus after SE that was associated mainly with M1 MG/MΦ through a MyD88-dependent pathway. Inhibition of MyD88 modulated MG/MΦ polarization, which may in turn suppress inflammation and gliosis and thus protect hippocampal neurons from apoptosis after SE. We also showed that inhibition of MyD88 upregulated GLT-1 expression and reduced NR1 expression in the hippocampus of SE mice 3 days after SE, which would be expected to improve glutamate transport and reduced postsynaptic excitatory effect. These findings shed light on how MG/MΦ polarization contributes to the pathological and molecular changes of the microenvironment of the epileptic brain.

To our knowledge, this study provides the first morphological evidence in the mouse brain documenting MG/MΦ polarization after SE in an epilepsy model. We provide new evidence on the progression of the neuroinflammation process in the hippocampus after a single SE event. We showed that M1 MG/MΦ were dramatically increased through the MyD88-dependent pathway in the hippocampus after SE and were retained in the chronic phase (28 days after SE). Because M1 MG/MΦ produce proinflammatory cytokines that induce an increase in excitatory synaptic transmission [34], our findings suggest that M1/M2 phenotype determination could be modulated via MyD88, which may provide a target for supporting microenvironment maintenance in the hippocampus after SE.

### Morphological Evidence for M1 MG/MΦ Response

The classical M1 polarization marker iNOS catalyzes NO synthesis. When MG/MΦ are stimulated by cytokines or microbe components, iNOS is induced to synthesize excess NO, resulting in a series of stress reactions. Recent studies showing that antiepileptic drugs reduce iNOS expression in animals [35, 36] suggest that iNOS participates in driving epilepsy.

Here, we demonstrated a continuous increase in iNOS-positive (M1) MG/MΦ in the hippocampus of mice after SE, and the increase remained predominant in the chronic phase. These data were consistent with a previous report showing sustained inflammation in the hippocampus after SE [37]. Dynamic observation suggested that the distribution of M1 MG/MΦ changed over time. In the acute phase (1~3 days), they were observed mainly in the stratum lacunosum-moleculare of area CA1 and the DG. In this phase, many neurons died of excitotoxicity caused by seizures. These dead cells (including those that died by necrosis and apoptosis) and cell debris induced inflammation via innate immune receptors such as TLR4. In the chronic phase (14 days and beyond), these cells increased in number and were found mainly in the stratum oriens, stratum pyramidale, and stratum radiatum of areas CA1 and CA3, especially the stratum pyramidale. The distribution of M1 MG/MΦ may reflect the course of neuronal damage. More importantly, the sustained aggregation of M1 MG/MΦ altered the microenvironment in these areas by way of a hyperinflammatory reaction, leading to apparent apoptosis of pyramidal neurons, as evidenced by TUNEL and Hoechst staining. Here, we do not exclude neuronal necrosis and/or autophagy during this process. These observations suggest that M1 MG/MΦ cytotoxicity may contribute to hippocampal sclerosis, which is likely involved in the pathogenesis of epilepsy.

These findings also support previous reports indicating in different epilepsy models that microglia M1/M2 polarization plays crucial roles in the pathogenesis of epilepsy[23–25]. Although the details of M1/M2 polarization differ from model to model, all these studies show that M1 polarization is increased in the early stage after SE even before myoclonus symptoms, which is consistent with the findings in our study. M2 polarization has been observed in pilocarpine-induced SE [23, 25] and loss-of-function mutations in the cystatin B gene (CSTB) [24] but not in a kainite-induced SE model [23]. M2 microglia were not found to be predominant after pilocarpine-induced SE in our study, which may be due to variations in model establishment, selected time points, and methods used for identifying M1/M2 phenotypes. Nevertheless, the suggestions of our findings are consistent with the conclusions of these studies that MG/MΦ polarization after SE crucially affects the outcome of epilepsy through modulation of the neuroinflammatory environment.

In addition to M1/M2 marker expression, expression of the TLR4 ligand HMGB-1 reflects inflammation severity [38, 39]. Along with TLR4 and MyD88, HMGB-1 immunoreactivity was found mainly in MG/MΦ in the early post-SE inflammatory reaction, indicating that the early inflammatory reaction was mediated mainly by MG/MΦ rather than astrocytes. In the chronic phase, HMGB-1 was highly expressed in karyorrhectic pyramidal neurons, indicating that the inflammatory process after SE is continuous, progressive, and complicated in the chronic phase when multiple cell types are involved. Similarly, in a previous study, changes in inflammation were observed within 3 h of SE together with HMGB-1 expression in various CA1 cells, and HMGB-1 was observed to act as the TLR4 ligand, thereby playing a key role in the precipitation and recurrence of seizures [6]. Together, these findings suggest that SE-induced HMGB-1 may interact with TLR4 on MG/MΦ and thereby provoke continuous MG/MΦ activation in the hippocampus during the acute phase, with inflammation being sustained and becoming complicated and persistent.

### Astrocytic ARG-1 Upregulation in the Brains of Mice After SE

Another glial cell type involved in neuroinflammation is astrocytes. The role of reactive astrocytes in seizures is controversial. They release both pro- and anticonvulsant cytokines [35, 40]. In addition to participating in the inflammatory response, astrocytes play important roles in glutamate transportation, glutamine synthesis, and water/potassium balance after SE [41]. Here, we deduce from our finding that astrocytes may dampen the effect of iNOS in the acute phase after SE via upregulation of ARG-1.

ARG-1, which competes with iNOS for the breakdown of arginine [20, 42], has an anti-inflammatory and repair-facilitating influence [12]. In our study, Western blotting revealed upregulated ARG-1 expression in the hippocampus during the acute phase of SE. However, our IHC experiments showed that ARG-1 was expressed mainly in astrocytes rather than in MG/MΦ during this period. ARG-1-expressing astrocytes were found previously in an ischemia model as well as in an autoimmune encephalomyelitis model [43, 44], suggesting that ARG-1 in astrocytes may be a reactive response to pathological conditions.

Although ARG-1-positive astrocytes may respond to inflammatory damage after SE, their protective influence is apparently insufficient to overcome the inflammation, given that astrocytes in our model continued to express high levels of HMGB-1, resulting in gliosis. Such post-SE gliosis may aggravate the complexity of the inflammatory response and, ultimately, may facilitate the development of spontaneous seizures. More studies are needed to distinguish the post-SE stage-specific functions of astrocytes. Notwithstanding, our findings support the notion that the inflammatory environment in the brain may be determined by MG/MΦ polarization.

### MyD88 Affects SE Outcome by Favoring the M2 MG/MΦ Phenotype

Skewing MG/MΦ polarization toward M2 could be an option to relieve neuronal excitation-induced inflammation after SE. The MyD88-dependent pathway is triggered by ligand (e.g., damage-associated molecular pattern protein) activation of TLR4, which leads to activation and translocation of NF-κB and to proinflammatory cytokine production. Overactivation of this pathway produces a hostile microenvironment for neurons in the CNS. MyD88 has been associated with diverse functions in the brain, including central metabolic processes, cognitive functions, and motor functions [45–47], suggesting that MyD88 may have extensive regulatory roles in the CNS. Our observation of selective MyD88 expression on MG/MΦ in the hippocampi of mice after SE indicates a role for MyD88 in MG/MΦ but not in neurons or astrocytes. Inhibition of MyD88 by MIP suppressed M1 polarization and tended to promote M2 polarization, as indicated by a significantly increased number of ARG-1-immunopositive MG/MΦ in the hippocampi of SE mice. The increase in ARG-1 protein levels in the MIP group was not significant compared with the CP group, as shown by Western blot. This discrepancy may be because astrocytes expressed ARG-1 in response to SE in CP-treated mice. MIP treatment increased the enzyme expression in MG/MΦ, but this increase may not dramatically outweigh astrocytic (other cell types cannot be excluded) ARG-1 expression. For CD163, another M2 marker, labeled MG/MΦ were found to be increased in the hippocampi of MIP-treated SE mice, suggesting M2 polarization by MyD88 inhibition. The inflammatory response was thus downregulated by MyD88 inhibition in the acute phase, which protected hippocampal pyramidal neurons against apoptosis and inhibited gliosis in the chronic phase. These effects account for an improved microenvironment within the hippocampus after SE and thus may lead to relief of hippocampal sclerosis in the chronic phase in the case of epilepsy.

In addition to the tendency toward M2 polarization, MyD88 inhibition or deficiency reduced the occurrence of SE induced by pilocarpine in mice. The data from MyD88 inhibition (MIP-treated mice) are consistent with a previous report on pilocarpine-induced SE in rats [30]. Notably, the blunted occurrence of SE in MyD88^−/−^ mice relative to WT mice appeared more obvious than that in MIP treatment in comparison with CP treatment. A previous study on virus infection-induced seizures showed that MyD88 deficiency led to a nonsignificant reduction in the incidence of SE in mice infected with Theiler’s murine encephalomyelitis virus [48], whereas the effect of MyD88 deficiency itself on SE was not documented. Accordingly, as we found, genetic MyD88 deficiency was more effective than MIP injection at reducing hippocampal M1 MG/MΦ responses: M1 MG/MΦ were almost entirely absent from the MyD88-deficient hippocampus, where M2 MG/MΦ had accumulated, after SE.

MyD88 ablation has also been shown by some [45], but not others [46], to impair locomotor function. This inconsistency may be due to the different behavioral tests used. Regardless, it indicates that the influences of MyD88 deficiency on motor function are probably conditional. In our pilot experiments, we did not observe significant differences in locomotor function between MyD88 inhibition/ablation and control/WT animals either. On the other hand, those behavioral observations did not include convulsions which are specific to seizures. In the present study, the animals’ convulsions persisted for 50 min in SE, which is far different from voluntary, coordinated movements and explorative locomotion, as previously reported. Hence, the blunting of SE by MyD88 inhibition/ablation may be related to its effects on other responses rather than an influence on movement.

### MG/MΦ Polarization Affects SE Through Glutamate-Metabolism-Related Excitation of Hippocampal Neurons

When MG/MΦ polarization was skewed toward M2 via MyD88 inhibition/deficiency, glutamate transportation was also changed in the hippocampi of SE mice. NMDA receptors play critical roles in epileptic excitotoxicity, and NMDA receptor antagonists have been used to treat refractory seizures [49, 50]. NMDA receptor subunit composition has been reported to be altered in epilepsy models [6, 41–53]. In addition, it has been reported that iNOS acts on mature neurons indirectly, increasing NMDA receptor expression [54]. Increased NMDA receptor expression has also been reported to increase iNOS levels [55], and inhibition of iNOS has been shown to reduce glutamate release [56]. Together, these studies suggest that iNOS enhances the neurotoxic effects of glutamate and thus may form part of a vicious cycle.

Here, we found that M1 MG/MΦ, along with pyramidal neurons, continuously expressed iNOS in area CA1 and area CA3 after SE, together with increased expression of glutamate receptor subtypes NR1 and NR2b. Consistent with previous studies [6, 52, 53], our findings indicate that M1 MG/MΦ polarization augments glutamate toxicity, which may lead to apoptosis (and/or other types of cell death) of pyramidal neurons and extensive gliosis, which in turn may underlie, at least in part, the potential occurrence of spontaneous seizures in the chronic phase.

On the other hand, both astrocytic and neuronal GLT-1 have been reported to protect against fatal epilepsy [57, 58], and reduction of glutamate toxicity has been considered to be an effective treatment for epilepsy. We observed that MyD88 inhibition increased both astrocytic and neuronal GLT-1 expression and that GLT-1-positive astrocytes gathered in the DG and in area CA3 during the acute phase. In addition, MyD88 inhibition reduced the expression of NR1, which was upregulated by SE. A similar inhibition of the NMDA receptor was reported in a previous study on SE rats treated with MyD88 inhibitor [30]. These processes may be achieved indirectly by reducing iNOS expression in M1 MG/MΦ. Together, these results suggest that MyD88 inhibition may downregulate glutamate efficiency and transport and may thus reduce glutamate neurotoxicity, thus helping to protect the CNS microenvironment after SE and likely helping to reduce the occurrence of spontaneous seizures.

It should be noted that although MyD88 is expressed mainly in MG/MΦ, it is also expressed in other cell types in the CNS, such as neurons [59] and astrocytes. Thus, possible effects of MyD88 inhibition or ablation on other cell types in the present study cannot be excluded. Shen and colleagues [60] documented that astrocytic MyD88 ablation reduced LPS-induced neuronal excitatory synaptogenesis in young mice and culture. However, in adults, evidence is lacking on whether MyD88 still works in astrocytes to enhance inflammatory stimulation-induced neuronal excitation. Nevertheless, based on our observation that most of the MyD88 protein expression observed in the hippocampus overlapped with expression of iba-1, an MG/MΦ marker, in the acute phase after SE, the protective effect of MyD88 inhibition on SE animals should be attributed predominantly to alternative MG/MΦ activation in this scenario.

## Conclusion

After SE, augmented M1 polarization of MG/MΦ persists into the chronic phase, and this increase is associated with a deleterious (proinflammatory) microenvironment in the brain. MyD88 inhibition or deficiency is found to skew MG/MΦ polarization from the M1 to the M2 phenotype and increases the expression of glutamate transporter proteins, eventually improving the pathological outcome and neuronal protection in the hippocampus. Our data suggest that MyD88 may represent a potential target for epilepsy treatment owing to its ability to affect MG/MΦ polarization.

## Electronic Supplementary Material

Supplementary Fig. 1 Hippocampal distribution of TLR4-immunoreactive MG/MΦ. (A1-A3) Few activated MG/MΦ and hardly any TLR4-positive cells in the DG (A1), CA1 (A2), and CA3 (A3) of the control group. Increased numbers of TLR4-positive MG/MΦ 1 d (B1-B3) and 3 d (C1-C3) after SE. (D1-D3) Higher magnification of the boxes in C1-C3. Arrows indicate TLR4-immunolabeled MG/MΦ. Arrowheads point to TLR4-positive, iba-1-negative cells with astrocytic morphology. The inset of (D2) shows a high-magnification view of TLR4-iba-1-colocalized cells. *Scale bars*: A1–C3, 100 μm; D1–D3, 50 μm; D2 (inset), 12.5 μm. (PNG 11729 kb)

High Resolution image (TIF 16156 kb)

Supplementary Fig. 2 Distribution of iNOS-immunolabeled MG/MΦ in the hippocampi of mice 3 d after SE. (A1-A3) In the control group, there were hardly any iNOS-iba-1 co-labeled cells in the DG, CA1, or CA3. (B1-B3) Significantly increased M1 MG/MΦ 3 d after SE. (C1-C3) Higher magnification of the boxes in B1-B3. Arrows point to iNOS-iba-1 double-labeled cells. The inset of (C3) shows high magnification of M1 MG/MΦ. (D) Summary of quantitative analysis of iba-1/iNOS double-positive cells in hippocampi of the control group and 3 d group (means ± s.e.m., *n* = 3). *Scale bars*: A1–B3, 100 μm; C1–C3, 50 μm; C3 (inset), 12.5 μm. (PNG 8825 kb)

High Resolution image (TIF 24553 kb)

Supplementary Fig. 3 Immunohistochemical labeling for ARG-1, iNOS, MyD88, and MR along with NeuN, iba-1, and GFAP in the CA1 3 d after SE. MyD88 immunoreactivity was localized mainly in activated MG/MΦ, while ARG-1 and MR immunostaining appeared mainly in astrocytes. (A1-A3) ARG-1 was strongly colocalized with GFAP. (B1-B3) Immunofluorescent labeling for iNOS with NeuN, iba-1, and GFAP, respectively, showing iNOS colocalization with the MG/MΦ marker iba-1. (C1-C3) MyD88 immunostaining with NeuN, iba-1, and GFAP; MyD88 was observed mainly in iba-1-positive cells. (D1-D3) Immunolabeling of MR with NeuN, F4/80, and GFAP shows MR immunoreactivity mainly colocalized with GFAP. Arrows indicate double-labeled cells. Arrowheads indicate single-positive cells. *Scale bars*: A1–D3, 50 μm. (E-H) Percentages of cells staining positive for ARG-1, iNOS, MyD88, and MR among neurons, astrocytes, or MG/MΦ in CA1 at 3 d (means ± s.e.m., *n* = 3). (PNG 5919 kb)

High Resolution image (TIF 8576 kb)

Supplementary Fig. 4 Immunohistochemical labeling for CD163 and F4/80 in the hippocampi of the mice in the control group and 3 d after SE treatment with CP and MIP. Sections from the control group (A1, A2 and A3) show hardly any hippocampal cells stained positive for CD163 or F4/80. In the CP group, there were remarkable numbers of F4/80-positive cells in the DG (B1), CA1 (B2), and CA3 (B3) of the hippocampus, whereas CD163 labeling was very scarce. In the MIP group, CD163 and F4/80 double-labeled cells were distributed in all the subareas of the hippocampus (C1, C2 and C3). The insets show high magnification of the labeled cells in the area indicated by the arrows. (D) Quantification of CD163/F4/80 double-labeled cells in the subareas of the hippocampus in the control, CP and MIP groups (means ± s.e.m., *n* = 3). *Scale bars*: A1–C3, 100 μm; insets, 12.5 μm. (PNG 63224 kb)

High Resolution image (TIF 184776 kb)

Supplementary Fig. 5 Distribution of ARG-1 and GFAP double-labeled cells in the hippocampus. (A1-A3) Sections from the mice in the control group showed very few activated astrocytes and hardly any ARG-1 immunostaining in the DG, CA1, or CA3. (B1-B3) Three days after SE, there were remarkably increased numbers of ARG-1-positive astrocytes labeled with GFAP. (C1-C3) Higher magnification of the boxes in (B1-B3). The arrow indicates a strongly ARG-1-immunopositive cell. The inset of (C3) shows a high-magnification view of this cell. *Scale bars*: A1–B3, 100 μm; C1–C3, 50 μm; C3 (inset), 12.5 μm. (E) Quantification of GFAP/ARG-1 double-labeled cells in the subregions of the hippocampus in the control group and 3 d group (means ± s.e.m., n = 3). (PNG 9782 kb)

High Resolution image (TIF 24553 kb)

Supplementary Fig. 6 The distribution of ARG-1-positive MG/MΦ in the hippocampi of mice 28 d after SE with MyD88 inhibition treatment. Sections from the CP group (A1-A3) showing ARG-1 and iba-1 double staining in the DG, CA1, and CA3. Note that a number of iba-1-positive but ARG-1-negative cells were distributed in the CA1 and CA3; ARG-1-labeled cells were hardly ever MG/MΦ. (B1-B3) Higher magnification of the boxes in (A1-A3). (C1-C3) Increased ARG-1-positive MG/MΦ in the MIP group. (D1-D3) Higher magnification of the boxes in (C1-C3). Arrows show ARG-1 and iba-1 double-labeled cells. *Scale bars*: A1–A3, C1–C3, 100 μm; B1–B3, D1–D3, 50 μm. (PNG 12825 kb)

High Resolution image (TIF 18103 kb)

ESM 1 (PDF 1224 kb)

ESM 2 (PDF 1224 kb)
