# Supplementary figures and images for: Inhibition of MyD88 Signaling Skews Microglia/Macrophage Polarization and Attenuates Neuronal Apoptosis in the Hippocampus After Status Epilepticus in Mice
Source: Neurotherapeutics. 2018 Aug 15;15(4):1093–111. doi: 10.1007/s13311-018-0653-0 (PMC6277303; doi:10.1007/s13311-018-0653-0)

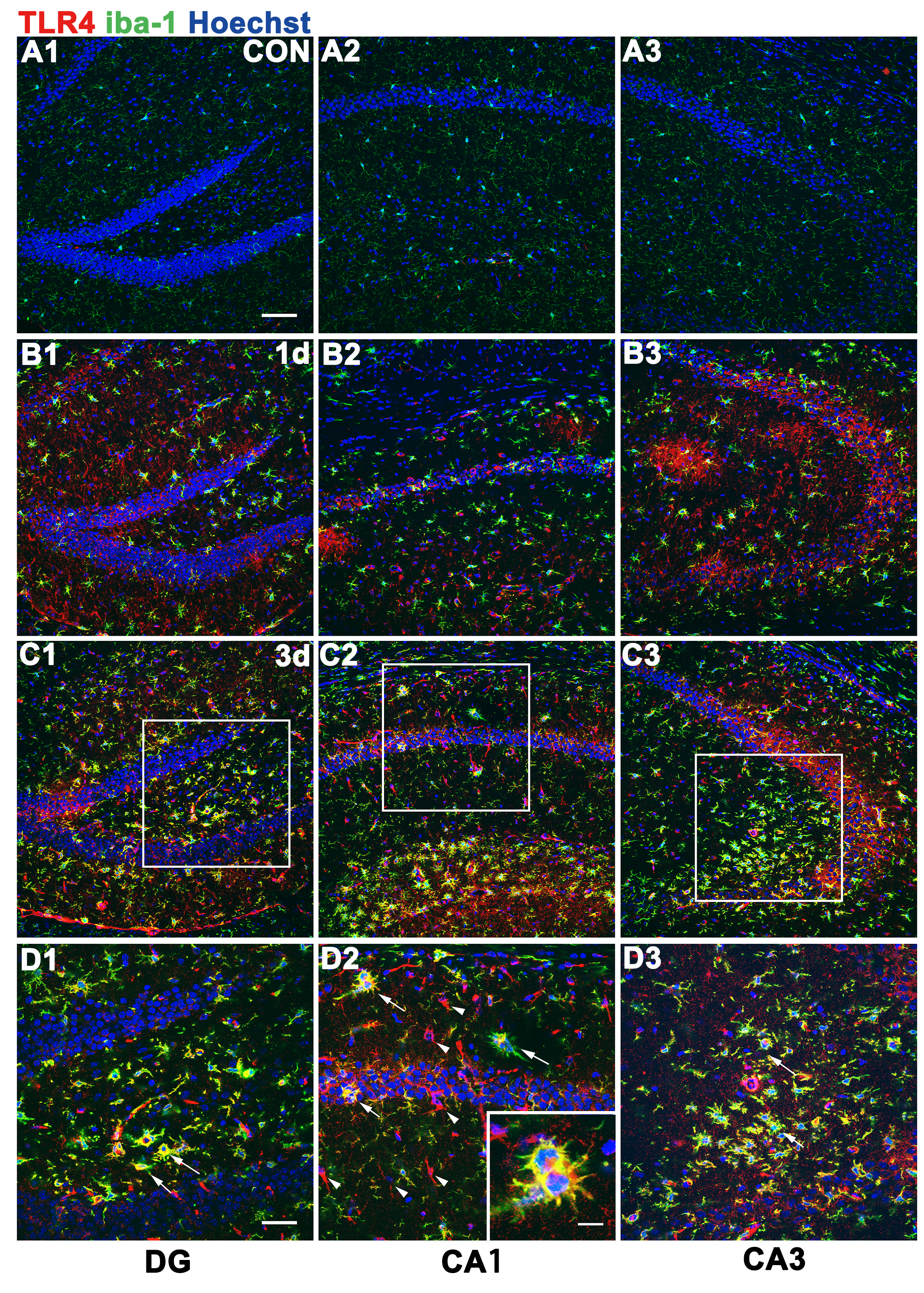

Supplement: Supplementary file 1 — Hippocampal distribution of TLR4-immunoreactive MG/MΦ. (A1-A3) Few activated MG/MΦ and hardly any TLR4-positive cells in the DG (A1), CA1 (A2), and CA3 (A3) of the control group. Increased numbers of TLR4-positive MG/MΦ 1 d (B1-B3) and 3 d (C1-C3) after SE. (D1-D3) Higher magnification of the boxes in C1-C3. Arrows indicate TLR4-immunolabeled MG/MΦ. Arrowheads point to TLR4-positive, iba-1-negative cells with astrocytic morphology. The inset of (D2) shows a high-magnification view of TLR4-iba-1-colocalized cells. Scale bars: A1–C3, 100 μm; D1–D3, 50 μm; D2 (inset), 12.5 μm. (PNG 11729 kb) [file 13311_2018_653_Fig10_ESM.png]

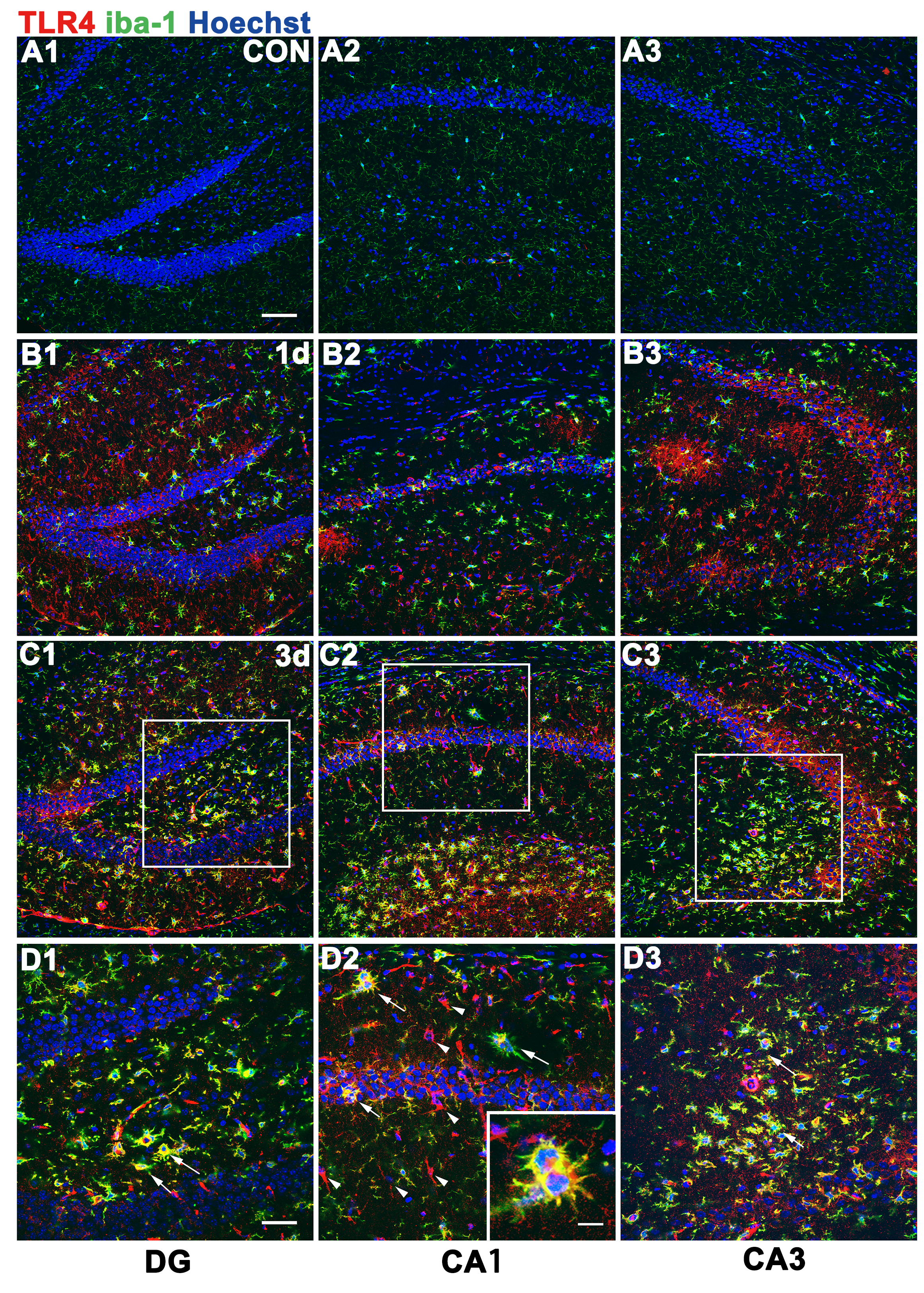

Supplement: Supplementary file 2 — High Resolution image (TIF 16156 kb) [file 13311_2018_653_MOESM1_ESM.tif]

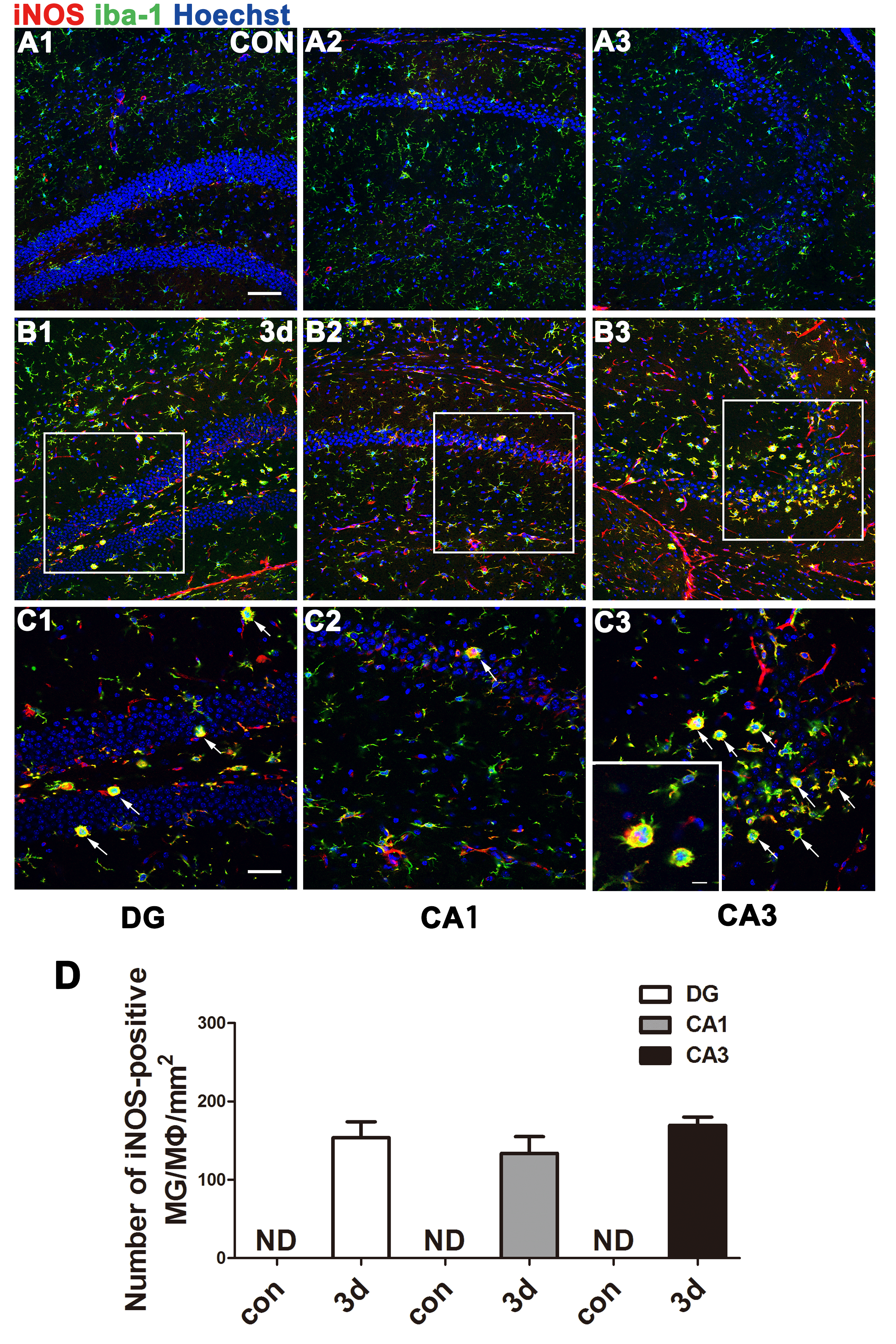

Supplement: Supplementary file 3 — Distribution of iNOS-immunolabeled MG/MΦ in the hippocampi of mice 3 d after SE. (A1-A3) In the control group, there were hardly any iNOS-iba-1 co-labeled cells in the DG, CA1, or CA3. (B1-B3) Significantly increased M1 MG/MΦ 3 d after SE. (C1-C3) Higher magnification of the boxes in B1-B3. Arrows point to iNOS-iba-1 double-labeled cells. The inset of (C3) shows high magnification of M1 MG/MΦ. (D) Summary of quantitative analysis of iba-1/iNOS double-positive cells in hippocampi of the control group and 3 d group (means ± s.e.m., n = 3). Scale bars: A1–B3, 100 μm; C1–C3, 50 μm; C3 (inset), 12.5 μm. (PNG 8825 kb) [file 13311_2018_653_Fig11_ESM.png]

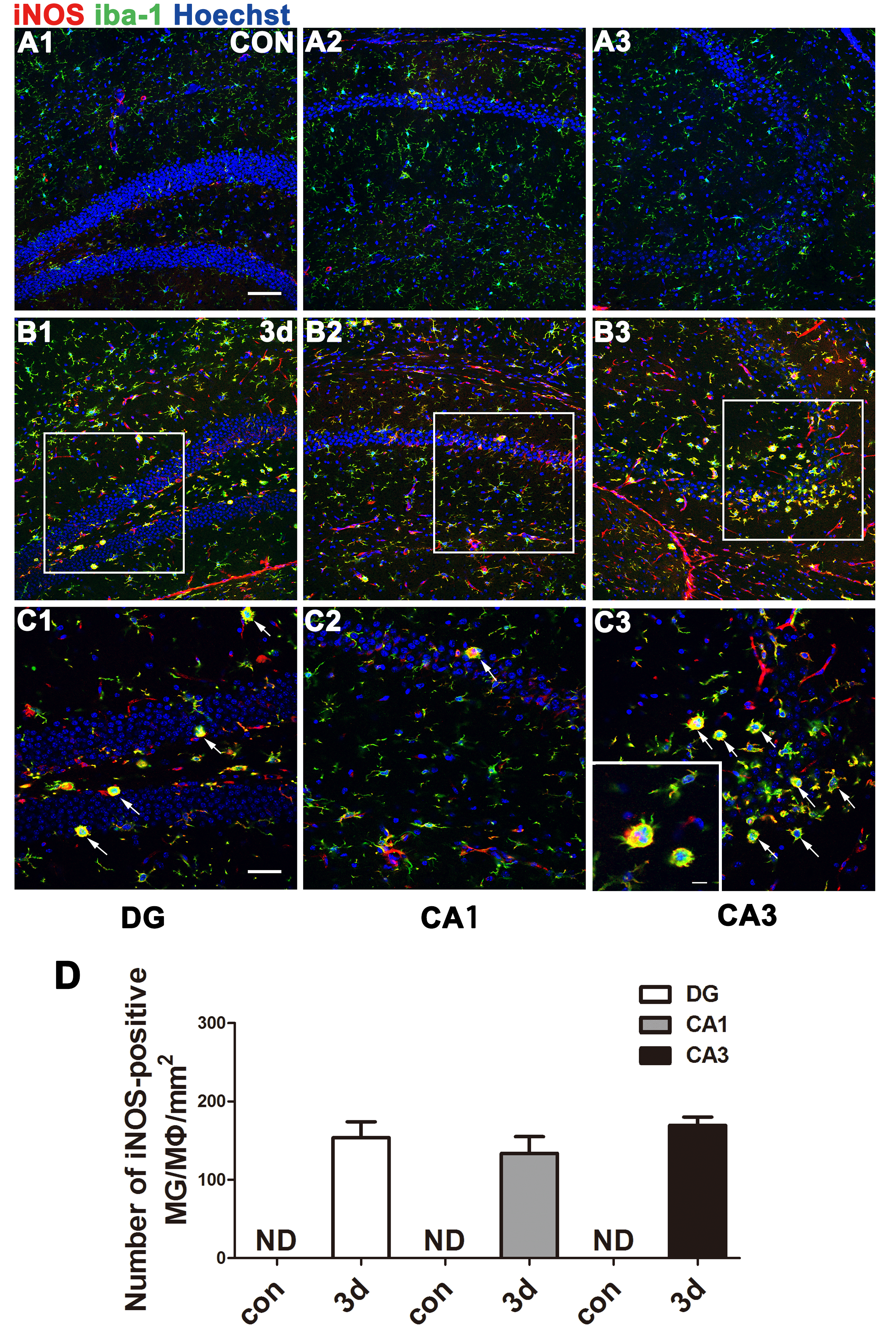

Supplement: Supplementary file 4 — High Resolution image (TIF 24553 kb) [file 13311_2018_653_MOESM2_ESM.tif]

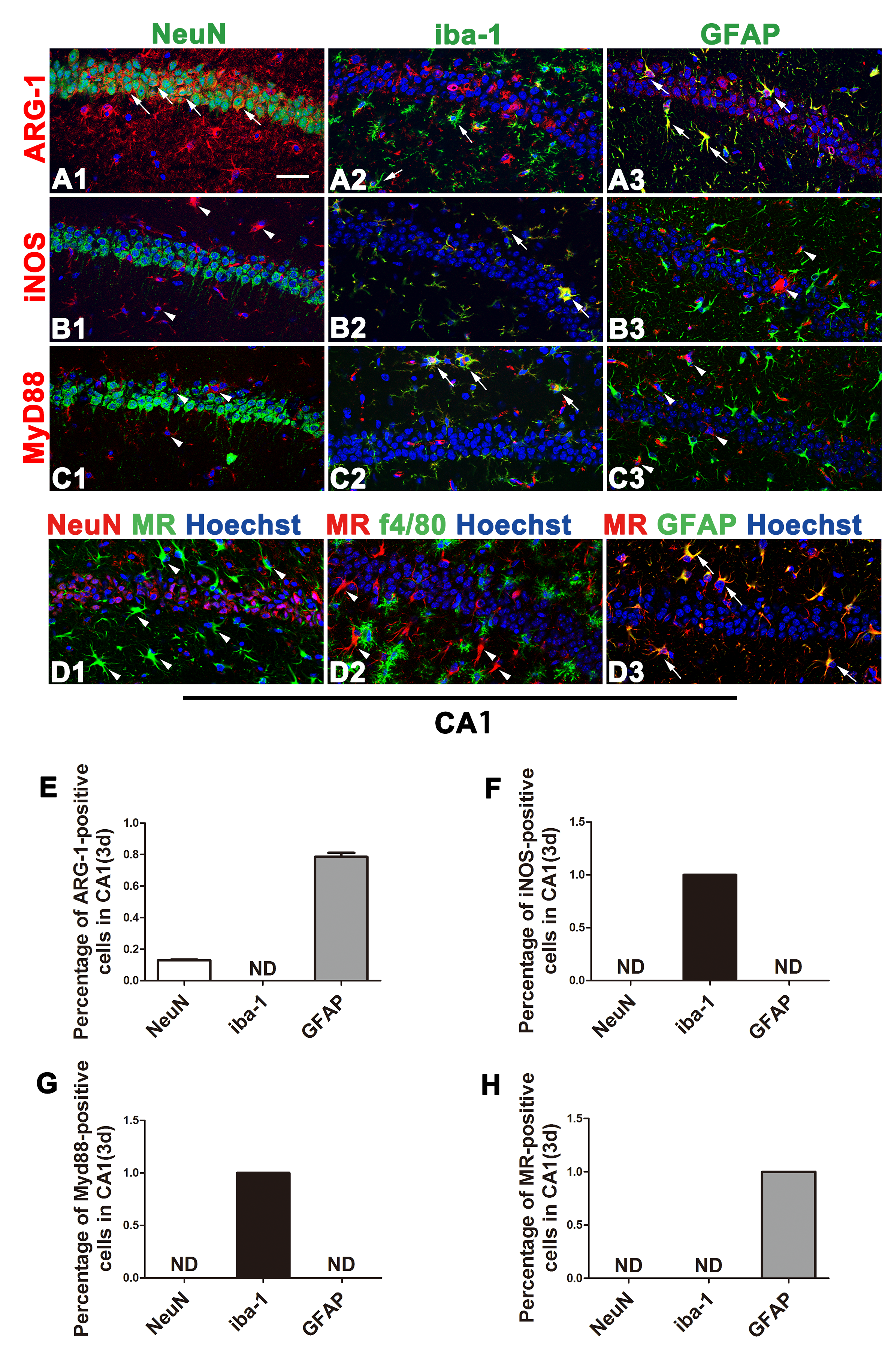

Supplement: Supplementary file 5 — Immunohistochemical labeling for ARG-1, iNOS, MyD88, and MR along with NeuN, iba-1, and GFAP in the CA1 3 d after SE. MyD88 immunoreactivity was localized mainly in activated MG/MΦ, while ARG-1 and MR immunostaining appeared mainly in astrocytes. (A1-A3) ARG-1 was strongly colocalized with GFAP. (B1-B3) Immunofluorescent labeling for iNOS with NeuN, iba-1, and GFAP, respectively, showing iNOS colocalization with the MG/MΦ marker iba-1. (C1-C3) MyD88 immunostaining with NeuN, iba-1, and GFAP; MyD88 was observed mainly in iba-1-positive cells. (D1-D3) Immunolabeling of MR with NeuN, F4/80, and GFAP shows MR immunoreactivity mainly colocalized with GFAP. Arrows indicate double-labeled cells. Arrowheads indicate single-positive cells. Scale bars: A1–D3, 50 μm. (E-H) Percentages of cells staining positive for ARG-1, iNOS, MyD88, and MR among neurons, astrocytes, or MG/MΦ in CA1 at 3 d (means ± s.e.m., n = 3). (PNG 5919 kb) [file 13311_2018_653_Fig12_ESM.png]

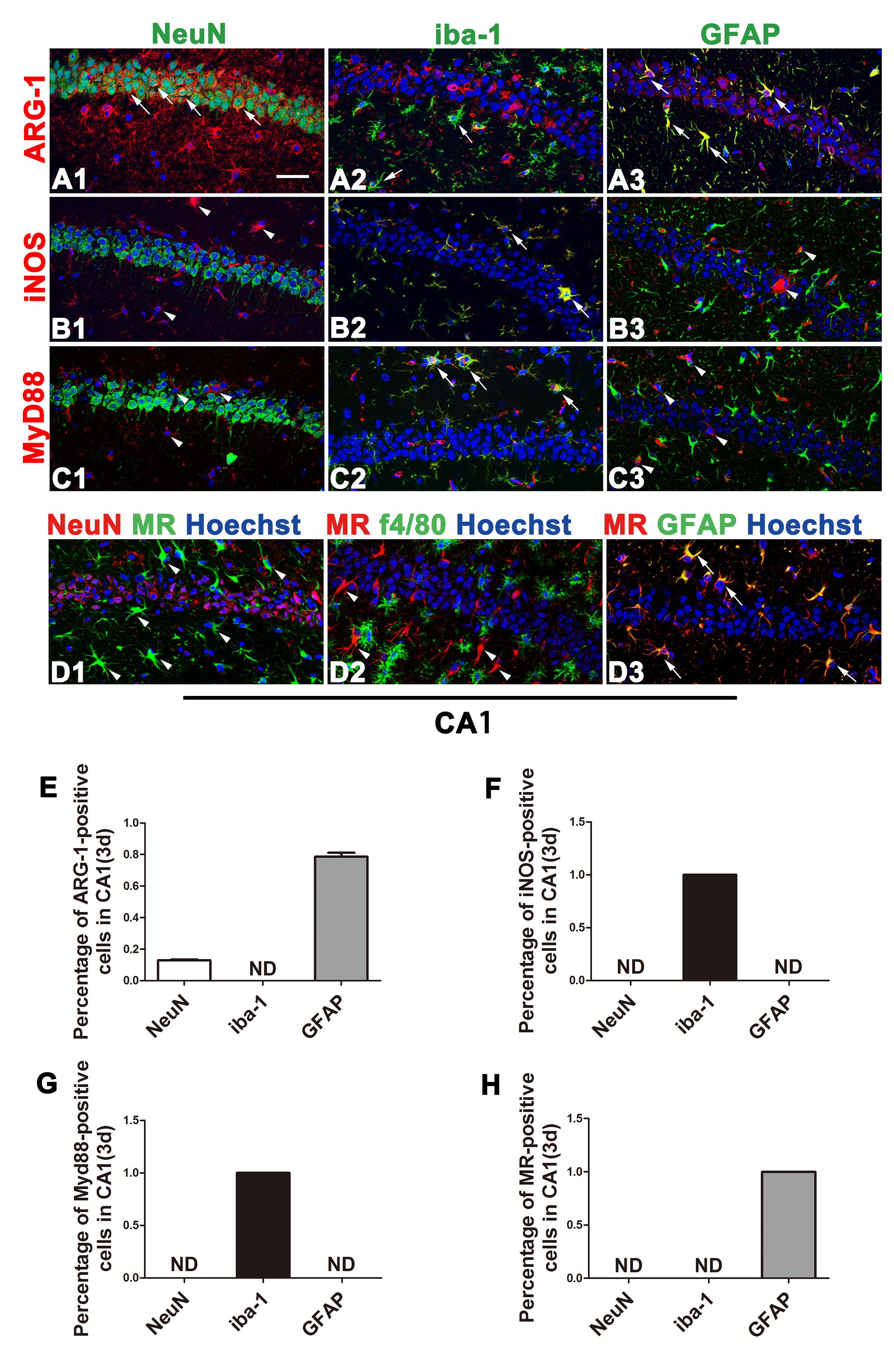

Supplement: Supplementary file 6 — High Resolution image (TIF 8576 kb) [file 13311_2018_653_MOESM3_ESM.tif]

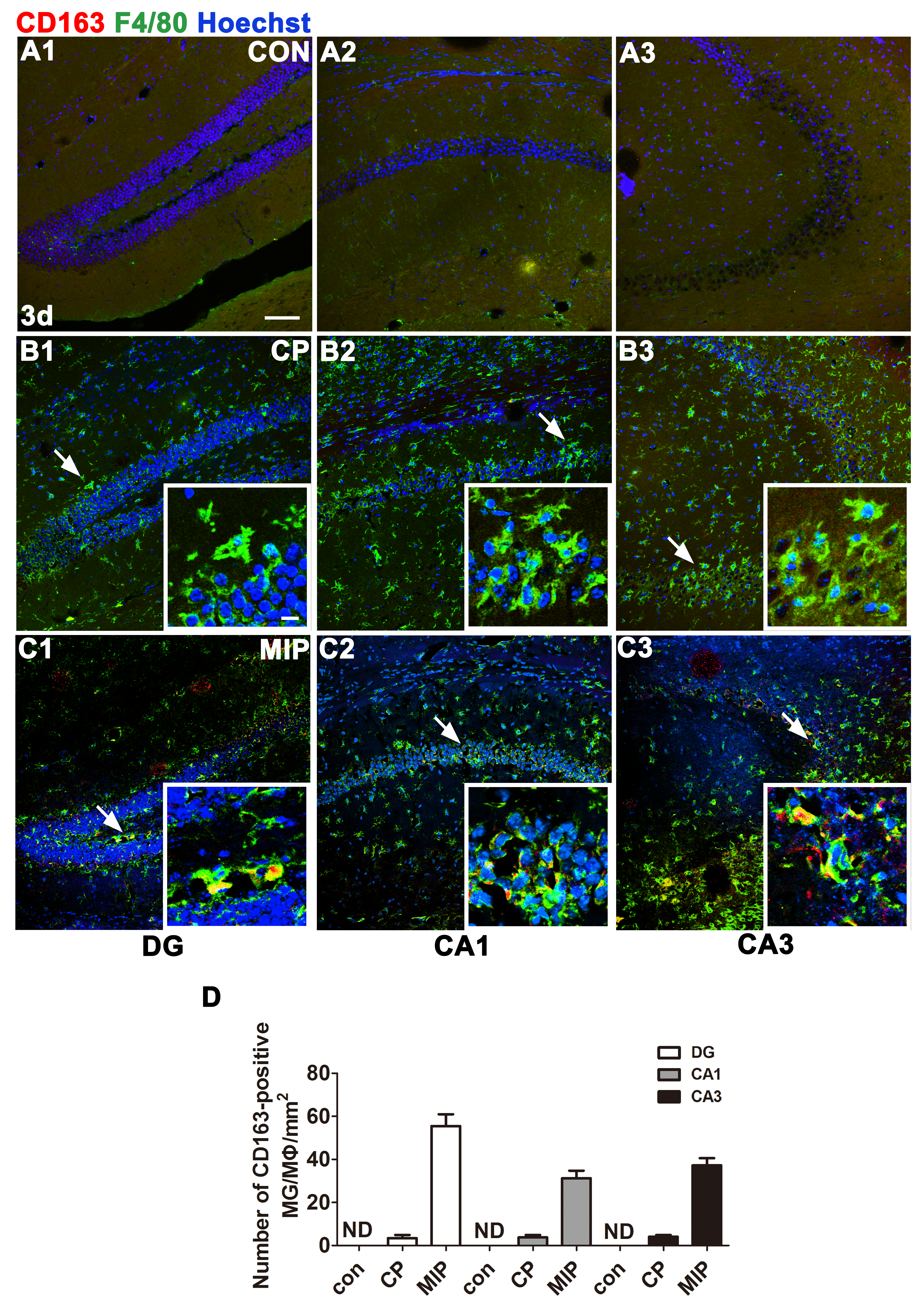

Supplement: Supplementary file 7 — Immunohistochemical labeling for CD163 and F4/80 in the hippocampi of the mice in the control group and 3 d after SE treatment with CP and MIP. Sections from the control group (A1, A2 and A3) show hardly any hippocampal cells stained positive for CD163 or F4/80. In the CP group, there were remarkable numbers of F4/80-positive cells in the DG (B1), CA1 (B2), and CA3 (B3) of the hippocampus, whereas CD163 labeling was very scarce. In the MIP group, CD163 and F4/80 double-labeled cells were distributed in all the subareas of the hippocampus (C1, C2 and C3). The insets show high magnification of the labeled cells in the area indicated by the arrows. (D) Quantification of CD163/F4/80 double-labeled cells in the subareas of the hippocampus in the control, CP and MIP groups (means ± s.e.m., n = 3). Scale bars: A1–C3, 100 μm; insets, 12.5 μm. (PNG 63224 kb) [file 13311_2018_653_Fig13_ESM.png]

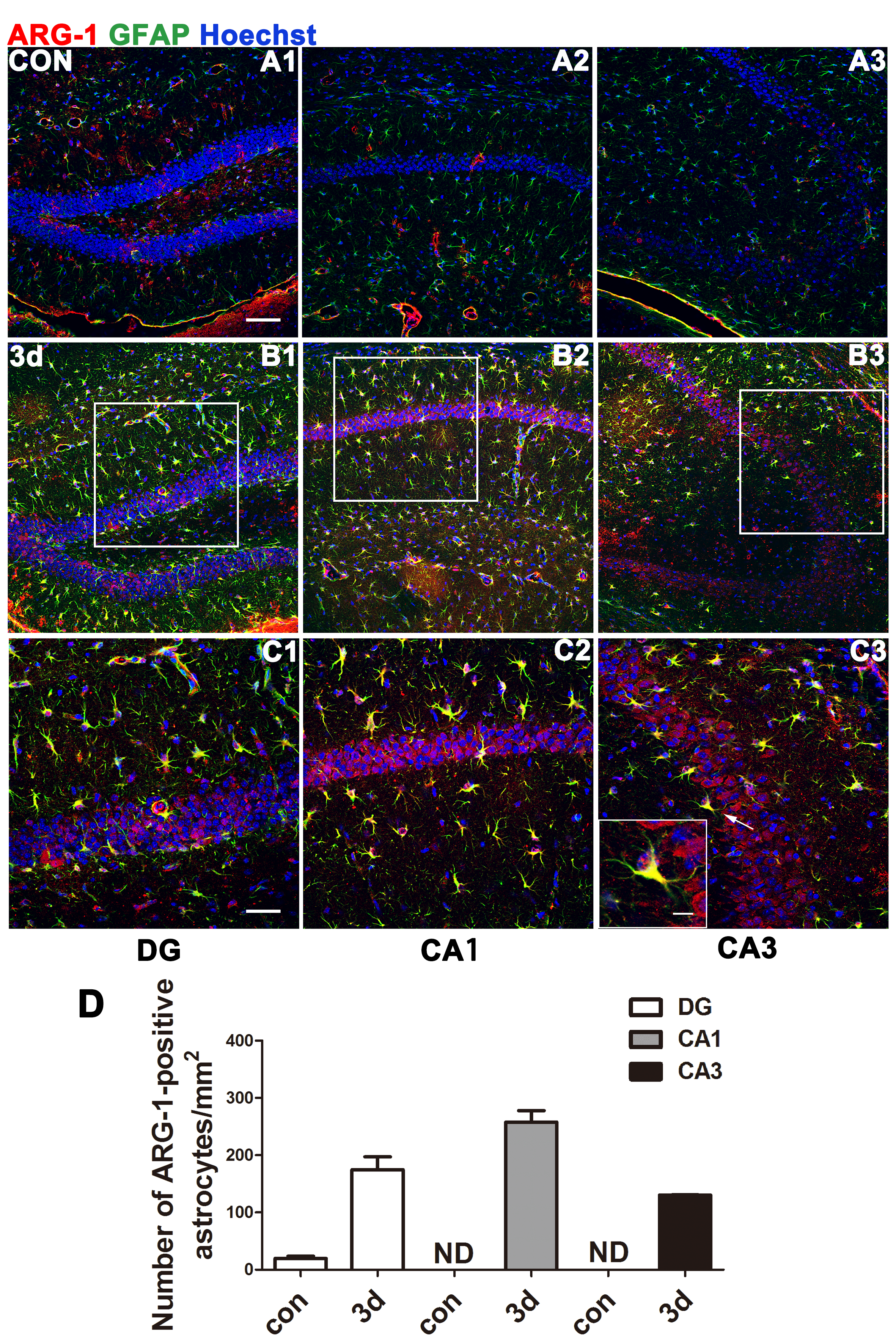

Supplement: Supplementary file 9 — Distribution of ARG-1 and GFAP double-labeled cells in the hippocampus. (A1-A3) Sections from the mice in the control group showed very few activated astrocytes and hardly any ARG-1 immunostaining in the DG, CA1, or CA3. (B1-B3) Three days after SE, there were remarkably increased numbers of ARG-1-positive astrocytes labeled with GFAP. (C1-C3) Higher magnification of the boxes in (B1-B3). The arrow indicates a strongly ARG-1-immunopositive cell. The inset of (C3) shows a high-magnification view of this cell. Scale bars: A1–B3, 100 μm; C1–C3, 50 μm; C3 (inset), 12.5 μm. (E) Quantification of GFAP/ARG-1 double-labeled cells in the subregions of the hippocampus in the control group and 3 d group (means ± s.e.m., n = 3). (PNG 9782 kb) [file 13311_2018_653_Fig14_ESM.png]

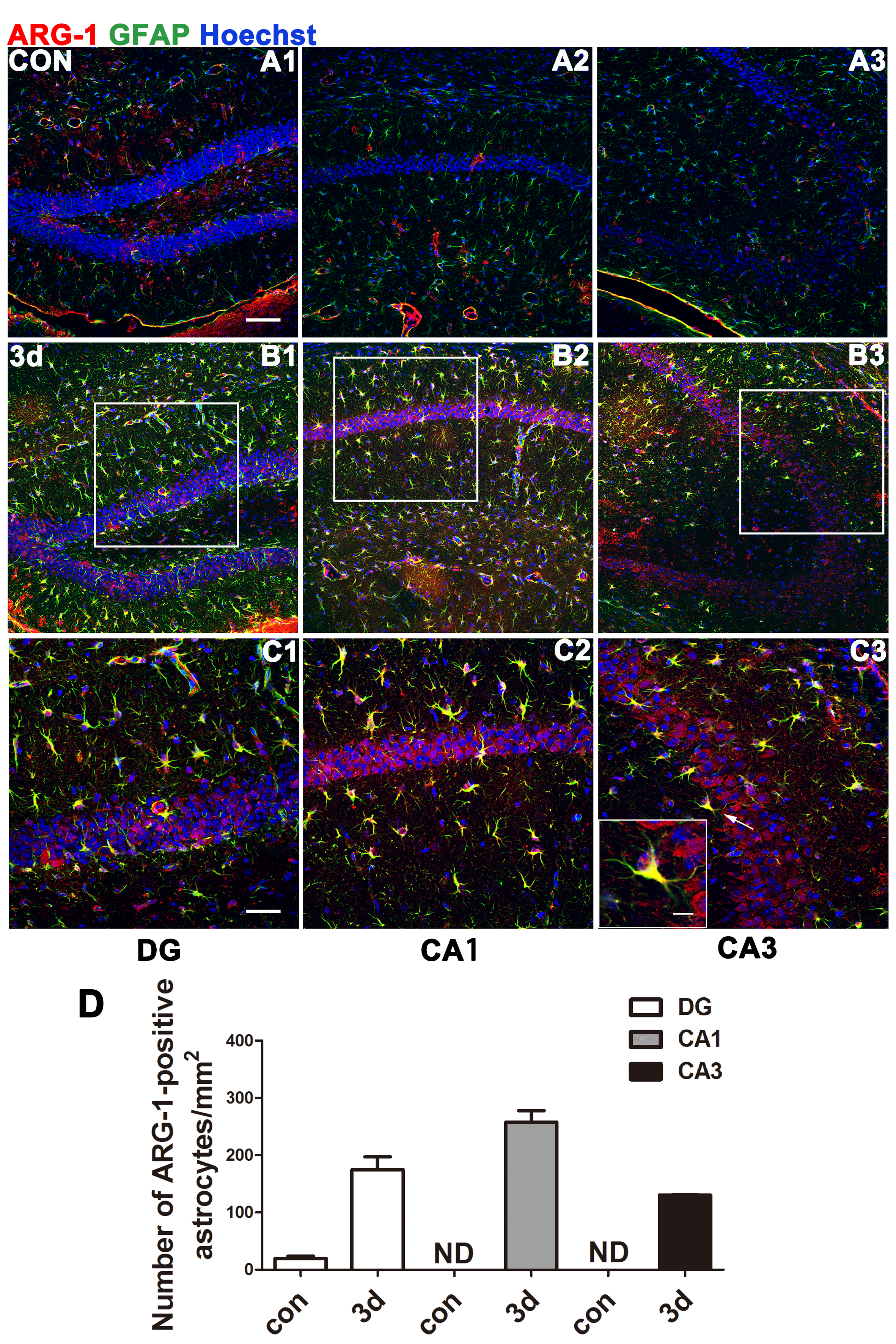

Supplement: Supplementary file 10 — High Resolution image (TIF 24553 kb) [file 13311_2018_653_MOESM5_ESM.tif]

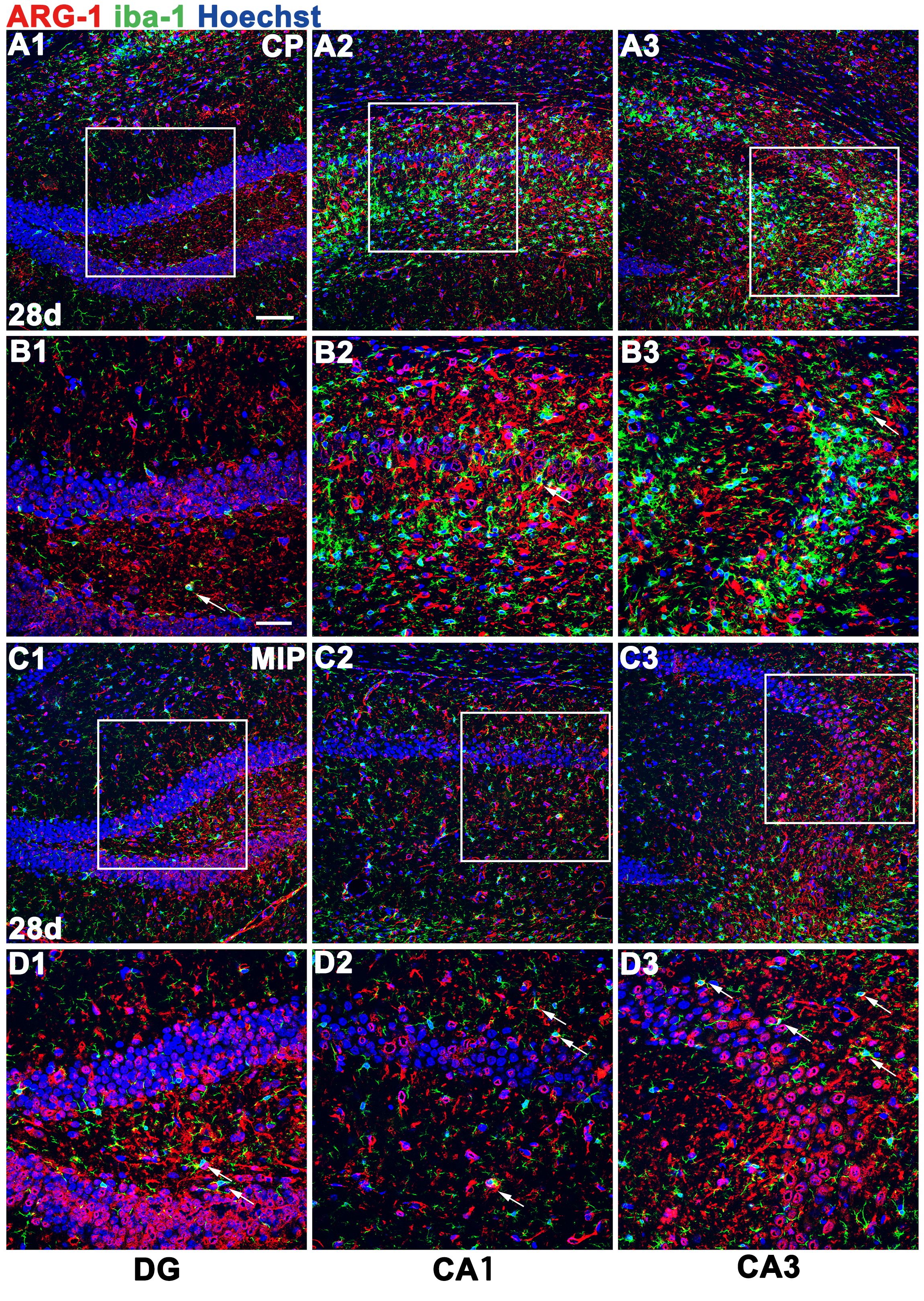

Supplement: Supplementary file 11 — The distribution of ARG-1-positive MG/MΦ in the hippocampi of mice 28 d after SE with MyD88 inhibition treatment. Sections from the CP group (A1-A3) showing ARG-1 and iba-1 double staining in the DG, CA1, and CA3. Note that a number of iba-1-positive but ARG-1-negative cells were distributed in the CA1 and CA3; ARG-1-labeled cells were hardly ever MG/MΦ. (B1-B3) Higher magnification of the boxes in (A1-A3). (C1-C3) Increased ARG-1-positive MG/MΦ in the MIP group. (D1-D3) Higher magnification of the boxes in (C1-C3). Arrows show ARG-1 and iba-1 double-labeled cells. Scale bars: A1–A3, C1–C3, 100 μm; B1–B3, D1–D3, 50 μm. (PNG 12825 kb) [file 13311_2018_653_Fig15_ESM.png]

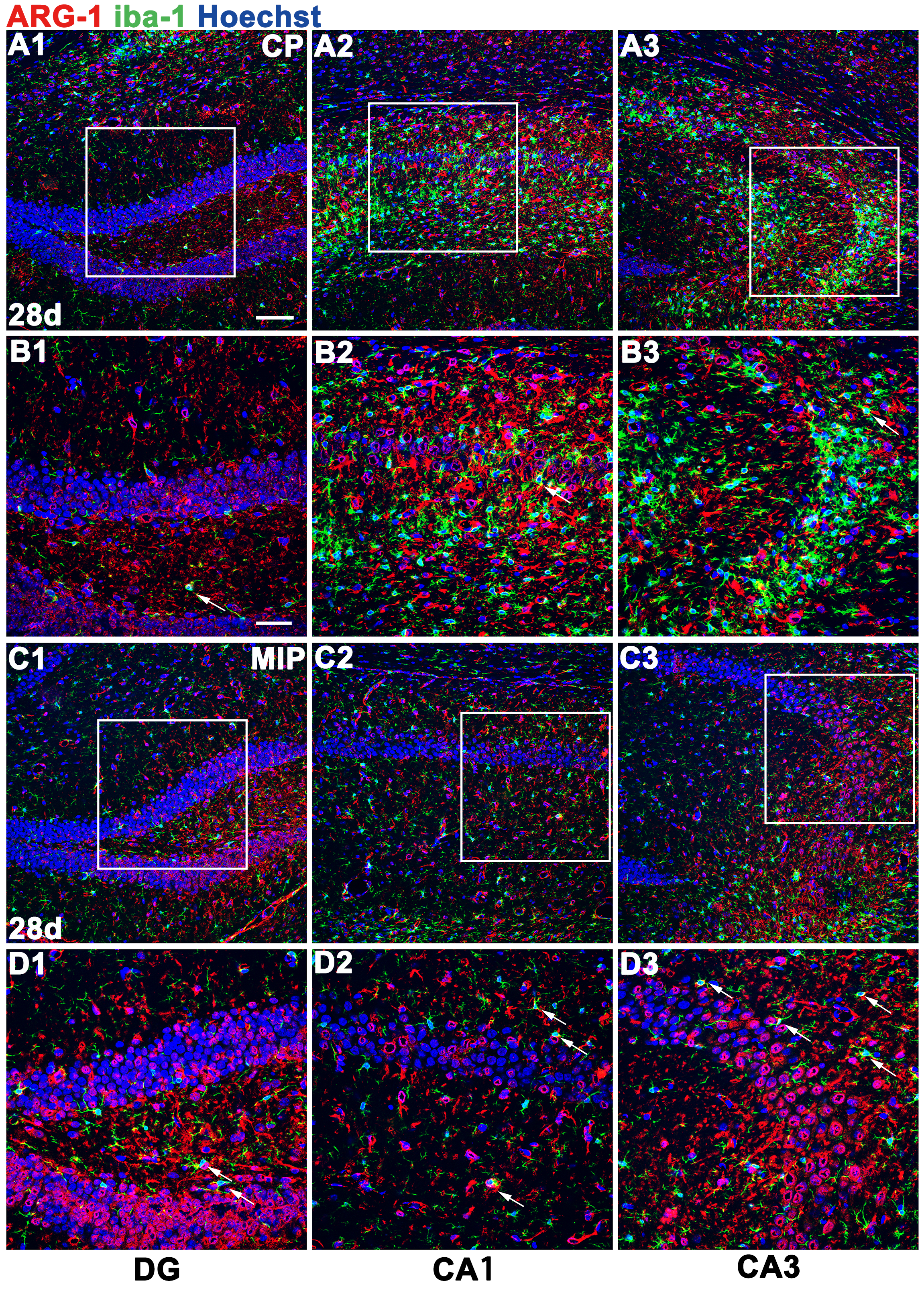

Supplement: Supplementary file 12 — High Resolution image (TIF 18103 kb) [file 13311_2018_653_MOESM6_ESM.tif]
